# Trends in the incidence of atrial fibrillation and the prescription of oral anticoagulants in adults with chronic obstructive pulmonary disease in UK primary care (2010–2022): a cohort study

**DOI:** 10.1016/j.eclinm.2026.104245

**Published:** 2026-09-28

**Authors:** Brianna Murray, Sarah Beradid, Pierre Ernst, Samy Suissa, Christel Renoux

**Affiliations:** a Department of Epidemiology, Biostatistics and Occupational Health, McGill University, Montreal, Canada; b Centre for Clinical Epidemiology, Lady Davis Institute for Medical Research, Jewish General Hospital, Montreal, Canada; c Department of Medicine, McGill University, Montreal, Canada; d Department of Neurology and Neurosurgery, McGill University, Montreal, Canada

**Keywords:** Chronic obstructive pulmonary disease, Atrial fibrillation, Oral anticoagulants, Time trends, UK

## Abstract

**Background:**

Understanding the burden of atrial fibrillation (AF) and oral anticoagulant (OAC) use in patients with chronic obstructive pulmonary disease (COPD) is important for stroke prevention in this high-risk population. We assessed trends in AF incidence and OAC initiation among patients with COPD in UK primary care from 2010 to 2022.

**Methods:**

We assembled a cohort of all patients at least 50 years of age who contributed data to the UK Clinical Practice Research Datalink between January 1, 2010, and December 31, 2022, who had a physician diagnosis of COPD before or during the study period. Patients were followed-up until AF diagnosis, study end date (December 2022), death, or transfer out of practice. We estimated annual incidence rates of AF and subsequent OAC initiation using Poisson regression, stratified by age, sex, ethnicity, and COPD severity. We assessed predictors of AF and OAC initiation using Cox proportional hazards regression models.

**Findings:**

444,668 patients with COPD were included (mean age 70 years, 51.8% male), contributing 2,284,799 person-years of follow-up. 37,645 (8.5%) patients were diagnosed with AF during the study period. The incidence of AF increased from 13.8 per 1000 person-years in 2010 to 19.3 in 2022 [rate ratio (RR) 1.29; 95% confidence interval (CI) 1.07–1.56], and was higher in males, White individuals, those over 80 years, and patients with severe COPD. Only 23,094 patients (66.3%) were prescribed an OAC in the year following AF diagnosis, though OAC use increased 2.9-fold during this period (RR 2.91; 95% CI 2.33–3.65). OAC initiation remained lowest among patients aged 50–59 and ≥90 years, and non-White individuals. OAC use was also up to 25.0% (adjusted hazard ratio 0.75; 95% CI 0.69, 0.81) less prevalent among those with severe COPD.

**Interpretation:**

AF incidence and OAC initiation increased among patients with COPD in UK primary care over 2010–2022. However, OAC use remained suboptimal, especially among older patients, non-White individuals, and those with severe COPD. These potential treatment gaps highlight the need for further research on stroke prevention strategies in this high-risk population.

**Funding:**

This research received no specific grant from any funding agency in the public, commercial or not-for-profit sectors.

**Research in context**

**Evidence before this study**

We searched PubMed for studies published from inception to August 30, 2025, using the search terms *(“chronic obstructive pulmonary disease”) AND (“atrial fibrillation”) AND (“incidence” OR “risk” OR “new onset”) AND (“time trends” OR “temporal trends” or “trends”).* Of 80 articles identified, only one study, conducted in Spain, examined trends in the incidence of atrial fibrillation (AF) in patients with chronic obstructive pulmonary disease (COPD). However, this study assessed AF-related hospitalizations, likely capturing a mixture of incident and prevalent cases, and did not report data beyond 2013. We also searched PubMed for articles published from inception to August 30, 2025, using the terms (“*chronic obstructive pulmonary disease*”) AND (“*atrial fibrillation*”) AND (“*oral anticoagulant”* OR *“anticoagulation*”). Of the 67 records identified, six observational studies and one systematic review and meta-analysis reported cross-sectional estimates of oral anticoagulant (OAC) use in patients with both COPD and AF. We found no real-world observational studies examining temporal trends or predictors of OAC prescribing for patients with COPD and AF.

**Added value of this study**

To our knowledge, this is the first population-based observational study to examine long-term temporal trends in the incidence of AF and OAC initiation among patients with COPD. Using nationwide UK primary care data from 2010 to 2022, we provide novel insights into the rising trends in AF incidence and OAC initiation in patients with COPD. We identify patient subgroups at greatest risk of AF, including those aged over 80 years, males, White individuals, and patients with more severe COPD. We also found that patients aged 90 years or older, non-White individuals, and those with more severe COPD were less likely to be prescribed an OAC, highlighting important disparities in care within this high-risk population.

**Implications of all the available evidence**

As AF becomes increasingly prevalent among individuals with COPD, OAC use may remain suboptimal, particularly among subgroups at highest thromboembolic risk. Our findings emphasize the need for more targeted, evidence-based guidelines for stroke prevention strategies for patients with coexisting COPD and AF. Additional studies evaluating the associations between OAC use and health outcomes in these patients, especially for those at greatest risk, may further contribute to optimizing stroke prevention strategies in this clinically complex population.

## Introduction

Chronic obstructive pulmonary disease (COPD) has a global prevalence of over 390 million and is currently the third leading cause of death globally.^1^ Atrial fibrillation (AF), the most common sustained arrythmia, affects over 52 million people worldwide, and approximately 13% of patients with COPD.^2^^,^^3^ A 2021 systematic review and meta-analysis estimated a 2-fold increased risk of AF in patients with versus those without COPD.^4^ The risk of AF has also been shown to increase with worsening forced expiratory volume in 1 s (FEV_1_), and with COPD exacerbation frequency, both important markers of COPD severity.^5^, ^6^, ^7^ However, no prior observational study has evaluated temporal trends in the incidence of AF in individuals with COPD. One study from Spain (2004–2013) assessed trends in the primary diagnosis of AF at hospital admission among individuals with COPD but did not report trends beyond 2013, captured both prevalent and incident cases, and did not consider outpatient and secondary in-hospital diagnoses.^8^

The occurrence of AF in patients with COPD is concerning, given that patients with comorbid COPD and AF have an increased risk of stroke compared to those with AF alone.^9^^,^^10^ Indeed, a recent cohort study has shown that patients with both COPD and AF have an approximate 1.6-fold higher risk of ischemic stroke compared to those with AF alone.^9^ Current management guidelines for patients with both AF and COPD suggest oral anticoagulant (OAC) treatment decisions for stroke and thromboembolism prevention be made independently of COPD status.^11^^,^^12^ However, evidence on OAC prescribing in patients with COPD is limited and inconsistent, with some studies reporting higher use, while others indicate lower use or no difference between those with and without COPD.^2^^,^^13^^,^^14^ Overall, OAC prescribing has increased significantly over the past decade among patients with AF, particularly following the 2011 approval of direct oral anticoagulants (DOACs) for stroke and thromboembolism prevention across the European Union and North America.^15^^,^^16^ Despite this progress, it remains unclear whether real-world OAC prescribing patterns are influenced by the presence of COPD.

Given the absence of specific guidelines for preventing stroke in patients with comorbid COPD and AF, as well as the unique clinical considerations in COPD, including inflammation, hypoxia, and polypharmacy, understanding temporal trends in the incidence of AF and OAC initiation is essential to optimizing stroke prevention strategies in this high-risk population.^12^ Therefore, the objective of our study was to assess trends in the incidence of AF and subsequent rates of OAC initiation in patients with COPD in primary care in the UK between 2010 and 2022.

## Methods

### Data source

We used the Clinical Practice Research Datalink (CPRD) Aurum database, a primary care database that contains medical records of over 50 million patients from more than 1800 general practices across England.^17^ Participating general practitioners routinely contribute anonymized patient-level medical records to the CPRD, including demographic data, lifestyle factors, diagnoses, prescriptions, referrals, immunizations, tests and results, using Egton Medical Information Systems (EMIS), Read, and Systematized Nomenclature of Medicine—Clinical Terms (SNOMED-CT) codes.^17^ The database is broadly representative of the general UK population in terms of geographical spread, deprivation, age, sex, and ethnicity.^17^ The information on medications and diagnoses has been validated and shown to be of high quality, including for studies of COPD and AF.^17^, ^18^, ^19^ We linked the CPRD to the Hospital Episode Statistics (HES) Admitted Patient Care (APC) data, which covers all hospital attendances to publicly funded hospitals in England, including details on admission and discharge dates, primary and secondary diagnoses, specialists seen, and procedures performed.^17^

### Study population

We assembled a cohort of all patients at least 50 years of age contributing data to the CPRD between 1 January 2010 and 31 December 2022, linked with HES APC data, with a physician diagnosis of COPD before or during the study period. Cohort entry was defined as either 1 January 2010, the date of COPD diagnosis, or the earliest date for which the patient had at least 12 months of medical information in the CPRD, whichever occurred later. We excluded patients below 50 years of age at COPD diagnosis to increase the likelihood of identifying patients with COPD rather than asthma. Patients with a diagnosis of AF prior to cohort entry, and those with less than 12 months of medical information in the CPRD were also excluded. Patients were followed until their first AF diagnosis, the study end date (31 December 2022), the patient's death or transfer out of practice, or the practice's last collection date, whichever occurred first. To assess OAC initiation in the year following AF diagnosis, we identified a sub-cohort comprising patients with COPD diagnosed with AF during follow-up. Patients with an OAC prescribed in the year before their first AF diagnosis were excluded from this sub-cohort. Patients were followed from the date of their first AF diagnosis until the earliest of the following: their first OAC prescription after AF diagnosis, one year post-AF diagnosis, death, transfer out of practice, the practice's last collection date, or the study end date (31 December 2022).

### Outcome

We identified all patients with an incident diagnosis of AF during follow-up using the relevant diagnostic codes. All OACs available in the UK over the course of study were identified. Vitamin K antagonists (VKAs) included warfarin, phenindione, and acenocoumerol; DOACs included dabigatran, rivaroxaban, apixaban, and edoxaban.

### Covariates

The following patient characteristics were identified at the time of AF diagnosis and separately at OAC initiation: age, sex, ethnicity, body mass index (BMI), smoking status, alcohol abuse, and comorbidities measured at any time before cohort entry: hypertension (defined as the presence of a diagnostic code or prescription for calcium channel blocker, angiotensin-converting enzyme inhibitor, angiotensin receptor blocker, or thiazide diuretic), diabetes, coronary artery disease (including myocardial infarction and ischemic heart disease), myocardial infarction, congestive heart failure, ischemic stroke/transient ischemic attack (TIA), systemic embolism, peripheral arterial disease, chronic kidney disease, liver disease, cancer (except non-melanoma skin cancer), history of bleeding, dementia, and obstructive sleep apnea. We also considered prescription of antiplatelet agents, antihypertensive drugs (calcium channel blockers, angiotensin-converting enzyme inhibitors, angiotensin receptor blockers, thiazides, and beta blockers), nonsteroidal anti-inflammatory drugs (NSAIDs), statins and lipid-lowering drugs in the prior year; and number of hospitalizations as a measure of healthcare utilization.

The CHA_2_DS_2_-VA score (congestive heart failure, hypertension, age 65–74 years, age ≥75 years, diabetes mellitus, prior stroke, TIA or thromboembolism, and vascular disease) was used to estimate the risk of stroke.^20^ Bleeding risk was assessed using the HAS-BLED score which consists of the following: hypertension, abnormal renal and/or liver function, stroke/TIA, history of bleeding, labile international normalized ratio (INR), age >65 years, and antiplatelet/NSAID use or alcohol abuse.^21^ Since INR measurements are not consistently reported in the CPRD, and new OAC users are unlikely to have an extensive history of INR results, we used a modified HAS-BLED score, without the INR element.

We also considered the severity of COPD at AF diagnosis and at OAC treatment initiation, measured by the number of moderate and severe COPD exacerbations, and hospitalization for pneumonia in the prior year. Moderate exacerbation was defined as the presence of a new prescription for prednisolone, while severe exacerbation was defined as a hospitalization for COPD (ICD-10 J41-J44). The most recent measure of blood eosinophil count, the number of bronchodilators [among long-acting muscarinic antagonists (LAMA) and long-acting beta agonists (LABA)], and prescriptions for other respiratory medications (short-acting inhaled beta agonists, short-acting antimuscarinics, inhaled corticosteroids, methylxanthines, and antibiotics for respiratory conditions) in the year prior to OAC initiation were also identified. Another measure of COPD severity was the most recent percent predicted FEV_1_ recording in the prior three years, for which the spirometry procedure quality in these general practices was found to be high.^22^ The percent predicted FEV_1_ measurement was calculated using the Global Lung Function Initiative global reference equation from the absolute FEV_1_ value, age, sex and height.^23^ We also considered dyspnea, measured by the modified Medical Research Council (mMRC) dyspnea scale, COPD assessment test (CAT) score, or presence of dyspnea symptoms, as previously defined.^24^ We used a missing category for variables that contained missing data (ethnicity, BMI, smoking status, dyspnea, and percent predicted FEV_1_). Ethnic categories (White, Black, Asian, other, and missing) were based on the higher-level classification used in the UK census, which aligns with the ethnicity classifications used in CPRD and HES APC records, as previously defined.^25^

### Statistical analysis

#### Trends in the incidence of AF among patients with COPD

We used a Poisson model to estimate the annual incidence rates of AF as the number of patients newly diagnosed with AF divided by the person-years of follow-up for all cohort members until their first AF diagnosis. We also estimated age- and sex-standardized annual rates of incident AF using the population structure of the cohort in the year 2022. We estimated the rate ratios (RRs) and 95% confidence intervals (CIs), adjusted for age and sex, comparing the annual incidence rates with those in 2010 and the year preceding each calendar year of study. The annual incidence rates were also stratified by age (50–59, 60–69, 70–79, 80–89, and ≥90 years), sex, ethnicity, geographic region of practice, and markers of COPD severity (most recent measure of percent predicted FEV_1_, severity of dyspnea, and history of moderate or severe COPD exacerbation in the prior year). Ethnicity was categorized as White or other for the stratified analyses, given the small number of individuals in the non-White ethnic subgroups. To account for changes in COPD severity over time, each marker of COPD severity was measured as time-varying throughout follow-up. All Poisson models included an overdispersion parameter to account for potential extra-Poisson variation. We also assessed predictors of AF incidence using a multivariable Cox proportional hazards regression model, conditioned on calendar year. We fitted the model with the following covariates: age, sex, ethnicity, BMI, smoking status, alcohol abuse, markers of COPD severity, dementia, cancer, obstructive sleep apnea, and the individual components of the CHA_2_DS_2_-VA and HAS-BLED scores.

#### Trends in OAC initiation among patients with COPD diagnosed with AF

These analyses were conducted using the sub-cohort of patients with COPD newly diagnosed with AF during the study period. We repeated the analyses mentioned above to estimate annual rates of OAC initiation overall, as well as separately for VKAs, DOACs, and individual DOACs. Annual OAC incidence rates were calculated as the number of patients newly prescribed an OAC, divided by the total person-months of follow-up for all cohort members until their first OAC prescription in the year following AF diagnosis. We stratified annual incidence rates by the CHA_2_DS_2_-VA score in males and females separately. We also calculated the proportion of prescriptions attributable to each OAC in each calendar year. Furthermore, we calculated the proportion of patients newly prescribed each OAC (VKA, apixaban, rivaroxaban, dabigatran, edoxaban) over the study period, stratified by age and sex. Additionally, we used a multivariable Cox proportional hazards regression model, conditioned on calendar year, to identify predictors of OAC initiation compared to no initiation. We also used a multivariable logistic regression model, conditioned on calendar year, to identify predictors of DOAC versus VKA initiation. We fitted these models with the following covariates: age, sex, ethnicity, markers of COPD severity, dementia, cancer, obstructive sleep apnea, and the individual components of the CHA_2_DS_2_-VA and HAS-BLED scores. Additionally, we assessed the percent of patients initiating beta blockers, except for sotalol, calcium channel blockers (diltiazem and verapamil), digitalis glycosides (digoxin and digitoxin), antiarrhythmic drugs (amiodarone, dronedarone, flecainide, and propafenone), and ablation procedures for AF in the year following AF diagnosis, stratified by age, sex, and COPD exacerbation in the prior year. We calculated the prevalence of LABA and LAMA use alone or in combination in the 3 months prior to and after AF diagnosis. Finally, for the logistic and Cox proportional hazards models, missing values were handled using a separate category for missingness.^26^ We conducted sensitivity analyses using multiple imputation to evaluate the impact of missing data on the adjusted odds ratios and hazard ratios.^27^ Multiple regression models were fitted with all covariates from the respective adjusted analysis model, the outcome, and auxiliary variables, including cardiovascular and respiratory medications, correlated with missingness or variables that contained missing data.^28^ Missing values were imputed for ethnicity, BMI, smoking status, dyspnea, and FEV_1_. We combined results from 20 imputed datasets using Rubin's rules.^29^ All analyses were performed using SAS 9.4 (SAS Institute Inc., Cary, NC).

### Ethics statement

The study protocol was approved by the CPRD Research Data Governance (No. 24_004368) and the Research Ethics Board of the Jewish General Hospital, Montreal, Canada, which waived the need for patient informed consent. The study was conducted in accordance with the Declaration of Helsinki.

### Role of the funding source

There was no specific funding source for this study.

## Results

### Trends in the incidence of AF among patients with COPD

After applying all selection criteria, 444,668 patients with COPD were included in the study cohort, contributing 2,284,799 person-years of follow-up (Figure S1). The mean age of patients was 70.1 years [standard deviation (SD): 9.9] and 230,310 (51.8%) were male. Within this cohort, 37,645 (8.5%) patients were diagnosed with AF during the study period. The mean age of patients diagnosed with AF was 77.8 years (SD: 8.3) and 21,784 (57.9%) were male (Table 1). At the time of AF diagnosis, cardiovascular disease, particularly hypertension (31,544 patients, 83.8%), diabetes mellitus (11,946, 31.7%), prior stroke (6629, 17.6%), chronic kidney disease (12,379, 32.9%), and history of bleeding (17,874, 47.5%) were common among patients newly diagnosed with AF.

**Table 1:** Baseline characteristics of cohort of all patients with chronic obstructive pulmonary disease, those newly diagnosed with atrial fibrillation (AF), and the sub-cohort of those prescribed an oral anticoagulant in the year following AF diagnosis between 2010 and 2022.

	Patients with COPD^a^ n = 444,668	Diagnosed with AF^b^, n = 37,645	Treated with an OAC^c^, n = 23,094
Age (years), mean (SD)	70.1 (9.9)	77.8 (8.3)	77.6 (7.9)
Sex			
Male	230,310 (51.8)	21,784 (57.9)	13,454 (58.3)
Female	214,358 (48.2)	15,861 (42.1)	9640 (41.7)
Ethnicity^d^			
White	403,068 (90.6)	36,028 (95.7)	22,079 (95.6)
Black	4208 (0.9)	242 (0.6)	139 (0.6)
Asian	8124 (1.8)	407 (1.1)	254 (1.1)
Other	4424 (1.0)	258 (0.7)	157 (0.7)
Missing	24,844 (5.6)	710 (1.9)	465 (2.0)
Body mass index			
< 30 kg/m^2^	286,837 (64.5)	23,492 (62.4)	14,092 (61.0)
≥ 30 kg/m^2^	117,601 (26.4)	12,618 (33.5)	8298 (35.9)
Missing	40,230 (9.0)	1535 (4.1)	704 (3.0)
Smoking			
Non-smoker	51,838 (11.7)	5206 (13.8)	3249 (14.1)
Ex-smoker	157,932 (35.5)	18,707 (49.7)	11,845 (51.3)
Smoker	230,381 (51.8)	13,435 (35.7)	7837 (33.9)
Missing	4517 (1.0)	297 (0.8)	163 (0.7)
Alcohol abuse	39,620 (8.9)	3809 (10.1)	2281 (9.9)
Moderate COPD exacerbation^e^^,^^h^	140,415 (31.6)	17,628 (46.8)	11,060 (47.9)
Hospitalization for COPD^h^			
0	421,451 (94.8)	32,783 (87.1)	19,879 (86.1)
1	18,998 (4.3)	3592 (9.5)	2434 (10.5)
≥ 2	4219 (1.0)	1270 (3.4)	781 (3.4)
Hospitalization for pneumonia^h^	58,397 (13.1)	10,123 (26.9)	5741 (24.9)
Severity of dyspnea^i^			
No/mild dyspnea	156,198 (35.1)	12,269 (32.6)	7845 (34.0)
Moderate/severe dyspnea	159,682 (35.9)	21,998 (58.4)	13,337 (57.8)
Missing	128,788 (29.0)	3378 (9.0)	1912 (8.3)
FEV_1_ (% predicted), mean (SD)^i^	63.1 (19.7)	60.4 (19.5)	61.4 (19.3)
< 50	84,430 (19.0)	9350 (24.8)	5456 (23.6)
50 –<80	169,255 (38.1)	15,493 (41.2)	9861 (42.7)
80–120	62,876 (14.1)	4799 (12.7)	3172 (13.7)
Missing	128,107 (28.8)	8003 (21.3)	4605 (19.9)
Blood eosinophil count cells/μl, mean (SD)	250.6 (208.2)	232.9 (215.8)	230.1 (213.5)
< 150	111,170 (25.0)	13,141 (34.9)	8316 (36.0)
150–300	158,820 (35.7)	14,205 (37.7)	8844 (38.3)
> 300	78,596 (17.7)	6577 (17.5)	4016 (17.4)
Missing	96,082 (21.6)	3722 (9.9)	1918 (8.3)
Respiratory medications^h^			
LABA	203,700 (45.8)	25,269 (67.1)	15,864 (68.7)
LAMA	156,949 (35.3)	21,889 (58.2)	14,027 (60.7)
Short-acting inhaled beta agonists	318,998 (71.7)	29,930 (79.5)	18,621 (80.6)
Short-acting antimuscarinics	41,492 (9.3)	2923 (7.8)	1578 (6.8)
Inhaled corticosteroids	225,680 (50.8)	24,634 (65.4)	15,163 (65.7)
Methylxanthines	13,832 (3.1)	2164 (5.8)	1218 (5.3)
Antibiotics for respiratory conditions	285,635 (64.2)	27,108 (72.0)	16,597 (71.9)
Comorbidities			
Hypertension	269,454 (60.6)	31,544 (83.8)	19,966 (86.5)
Diabetes	90,129 (20.3)	11,946 (31.7)	7687 (33.3)
Coronary artery disease^f^	99,338 (20.3)	15,250 (40.5)	9298 (40.3)
Myocardial infarction	45,356 (10.2)	8097 (21.5)	4869 (21.1)
Congestive heart failure	37,991 (8.5)	11,934 (31.7)	7628 (33.0)
Stroke/TIA	39,604 (8.9)	6629 (17.6)	4202 (18.2)
Systemic embolism	2309 (0.5)	334 (0.9)	187 (0.8)
Peripheral arterial disease	59,006 (13.3)	9644 (25.6)	5771 (25.0)
Chronic kidney disease	77,214 (17.4)	12,379 (32.9)	7346 (31.8)
Liver disease	23,355 (5.3)	2438 (6.5)	1427 (6.2)
Cancer^g^	72,905 (16.4)	9474 (25.2)	5485 (23.8)
History of bleeding	164,236 (36.9)	17,874 (47.5)	10,811 (46.8)
Dementia	20,276 (4.6)	3219 (8.6)	1600 (6.9)
Obstructive sleep apnea	11,449 (2.6)	1880 (5.0)	1244 (5.4)
Medications^h^			
Antiplatelet agents	135,981 (30.6)	17,968 (47.7)	11,988 (51.9)
Calcium channel blockers	115,449 (26.0)	14,460 (38.4)	9981 (43.2)
Angiotensin-converting enzyme inhibitors	115,293 (25.9)	13,228 (35.1)	9177 (39.7)
Angiotensin receptor blockers	48,869 (11.0)	6671 (17.7)	4450 (19.3)
Thiazides	60,873 (13.7)	6070 (16.1)	4027 (17.4)
Beta blockers	66,196 (14.9)	13,018 (34.6)	13,105 (56.8)
Nonsteroidal anti-inflammatory drugs	64,572 (14.5)	3750 (10.0)	2376 (10.3)
Statins or other lipid lowering drugs	201,803 (45.4)	22,216 (59.0)	14,623 (63.3)
Number of Hospitalizations^h^			
0	280,496 (63.1)	9949 (26.4)	6062 (26.3)
1	93,270 (21.0)	12,890 (34.2)	8023 (34.7)
≥ 2	70,902 (15.9)	14,806 (39.3)	9009 (39.0)
CHA_2_DS_2_-VA			
0	69,007 (15.5)	416 (1.1)	111 (0.5)
1	88,297 (19.9)	1865 (5.0)	948 (4.1)
≥ 2	287,364 (64.6)	35,364 (93.9)	22,035 (95.4)
Modified HAS-BLED			
≤ 2	234,875 (52.8)	9015 (24.0)	5075 (22.0)
> 2	209,793 (47.2)	28,630 (76.1)	18,019 (78.0)

Acronyms/abbreviations: CHA_2_DS_2_-VA, congestive heart failure, hypertension, age ≥75 years, diabetes mellitus, stroke/TIA, vascular disease, age 65–74 years; modified HAS-BLED, hypertension, abnormal renal and/or liver function, stroke/TIA, history of bleeding, age >65 years, antiplatelet/nonsteroidal anti-inflammatory drug use or alcohol abuse; COPD, chronic obstructive pulmonary disease; AF, atrial fibrillation; OAC, oral anticoagulant; SD, standard deviation; FEV_1_, forced expiratory volume in 1 s; LAMA, long-acting muscarinic antagonist; LABA, long-acting beta agonist; TIA, transient ischemic attack.

All values are expressed as n (%), unless otherwise specified.

a Measured at any time prior to cohort entry, unless otherwise specified.

b Measured at any time prior to initial AF diagnosis, unless otherwise specified.

c Measured at any time prior to initial OAC prescription in the year following AF diagnosis, unless otherwise specified.

d Ethnicity—Black: African, Caribbean, any other Black, African, or Caribbean background; Asian: Indian, Pakistani, Bangladeshi, Chinese, any other Asian; Other: Mixed, any other ethnic group.

e Moderate COPD exacerbation was defined as a prescription for prednisolone in the prior year.

f Coronary artery disease includes myocardial infarction and ischemic heart disease.

g Cancer includes all cancers except non-melanoma skin cancer.

h Measured in the prior year.

i Most recent measure in the prior 3 years.

Overall, the incidence rate of AF gradually increased over the study period, from 13.8 cases per 1000 person-years in 2010 to 19.3 cases per 1000 person-years in 2022, with a temporary drop in 2020 (Figure S2). Using the reference population of the study cohort in 2022, the annual age- and sex-standardized incidence rates were similar to the crude rates. The incidence rate of AF was 33% higher in 2022 compared to 2010 (RR: 1.33; 95% CI 1.10, 1.60), with similar RRs observed across strata of sex, age, ethnicity, and markers of COPD severity (Table 2). The incident RRs, adjusted for age and sex, comparing each calendar year's rate to that in the previous year, show an overall gradual yearly increase in AF incidence, except for a 22% drop in 2020 compared to 2019 (RR: 0.78; 95% CI 0.66–0.93) (Table S1).

**Table 2:** Incidence rate of atrial fibrillation per 1000 person-years in patients with chronic obstructive pulmonary disease in UK primary care, comparing 2022 with 2010, with 95% confidence intervals.

	2010 Rate^a^ (95% CI)	2022 Rate^a^ (95% CI)	RR (95% CI) 2022 versus 2010
Overall	13.82 (11.92, 16.02)	18.34 (16.3, 20.64)	1.33 (1.10, 1.60)
Age (years)			
50–59	3.42 (1.05, 11.11)	4.31 (1.55, 11.94)	1.26 (0.27, 5.98)
60–69	7.27 (4.99, 10.60)	7.96 (5.62, 11.27)	1.09 (0.65, 1.83)
70–79	14.78 (11.67, 18.72)	18.27 (15.16, 22.01)	1.24 (0.91, 1.67)
80–89	24.62 (19.47, 31.14)	34.80 (28.94, 41.86)	1.41 (1.05, 1.91)
≥90	28.99 (15.06, 55.81)	42.62 (27.35, 66.40)	1.47 (0.67, 3.24)
Sex			
Female	12.03 (9.55, 15.15)	15.45 (12.85, 18.57)	1.28 (0.96, 1.73)
Male	15.49 (12.78, 18.78)	21.26 (18.22, 24.80)	1.37 (1.07, 1.76)
Ethnicity^b^			
White	14.38 (12.39, 16.69)	19.65 (17.41, 22.18)	1.37 (1.13, 1.66)
Other^c^	7.03 (1.71, 28.88)	11.01 (4.67, 25.97)	1.57 (0.30, 8.18)
Dyspnea^b^			
no-mild	9.85 (7.79, 12.47)	13.35 (11.14, 16.00)	1.35 (1.01, 1.82)
moderate-severe	18.87 (15.10, 23.57)	24.64 (20.63, 29.42)	1.31 (0.98, 1.74)
Percent predicted FEV_1_^b^			
≥ 80%	9.92 (6.41, 15.36)	14.52 (10.17, 20.73)	1.46 (0.83, 2.57)
50–<80%	13.18 (10.84, 16.02)	18.95 (15.38, 23.35)	1.44 (1.08, 1.91)
< 50%	15.60 (12.56, 19.39)	19.46 (14.36, 26.38)	1.25 (0.86, 1.81)
Moderate-severe COPD exacerbation			
none	10.65 (8.69, 13.05)	15.20 (12.99, 17.79)	1.43 (1.10, 1.85)
≥1	19.90 (15.78, 25.11)	24.27 (20.01, 29.43)	1.22 (0.90, 1.65)
Severe COPD exacerbation			
none	12.17 (10.42, 14.23)	16.76 (12.84, 18.93)	1.38 (1.13, 1.68)
≥1	42.90 (22.65, 81.23)	55.83 (30.88, 101.00)	1.30 (0.54, 3.11)

Abbreviations: RR, rate ratio; CI, confidence interval; COPD, chronic obstructive pulmonary disease; FEV_1_, forced expiratory volume in 1 s.

a Crude incidence rate per 1000 person-years.

b Person-time and corresponding events were excluded for follow-up intervals with missing ethnicity [3.7% (15 person-years) of total person-time and 1.4% (28 events) of total events in 2010; 8.0% (35 person-years) of total person-time and 3.5% (111 events) of total events in 2022], dyspnea [14.9% (61 person-years) of total person-time and 11.9% (244 events) of total events in 2010; 11.6% (56 person-years) of total person-time and 9.2% (295 events) of total events in 2022], or severity classifications for FEV_1_ [19.4% (79 person-years) of total person-time and 21.2% (433 events) in 2010; 58.9% (281 person-years) of total person-time and 59.8% (1912 events) of total events in 2022].

c Other includes individuals identifying as Black African, Black Caribbean, any other Black, African, or Caribbean background, Indian, Pakistani, Bangladeshi, Chinese, any other Asian background, Mixed, or any other ethnic group.

Throughout the study period, incidence rates of AF were consistently higher in males than in females across all age groups and increased gradually with age (Fig. 1 and Figures S3-S4). Individuals of White ethnicity had higher annual incidence rates compared to those of other ethnic backgrounds (Figure S5). The trends were also similar across geographic region of practice (Figure S6). The incidence rates of AF were higher among individuals with more severe COPD, across all study years and regardless of the measure used (Fig. 2 and Figures S7-S9). These patterns remained consistent when stratified by age and sex (Fig. 3 and Figures S10-S12). Based on the Cox proportional hazards regression model, older patients, males, White individuals, and patients with obesity, cardiovascular disease, a history of stroke and alcohol abuse, chronic kidney disease, a history of bleeding, obstructive sleep apnea, and severe COPD were more likely to be diagnosed with AF (Table S2). These findings were consistent in a sensitivity analysis using imputed values for missing ethnicity, FEV_1_, dyspnea, and BMI (data not shown).

**Fig. 1:**
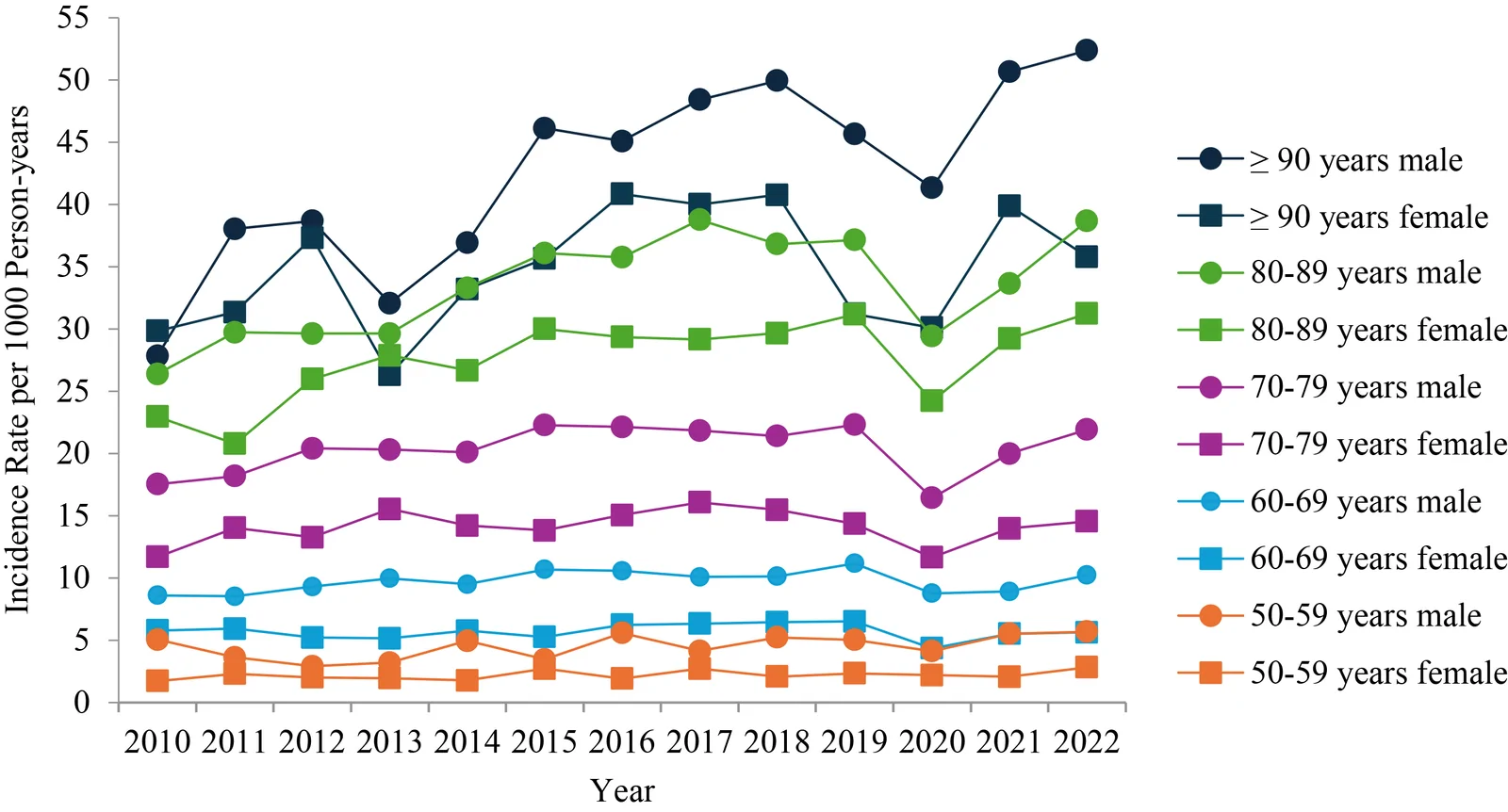
Crude incidence rates of atrial fibrillation per 1000 person-years, stratified by age and sex, in patients with chronic obstructive pulmonary disease in UK primary care from 2010 to 2022.

**Fig. 2:**
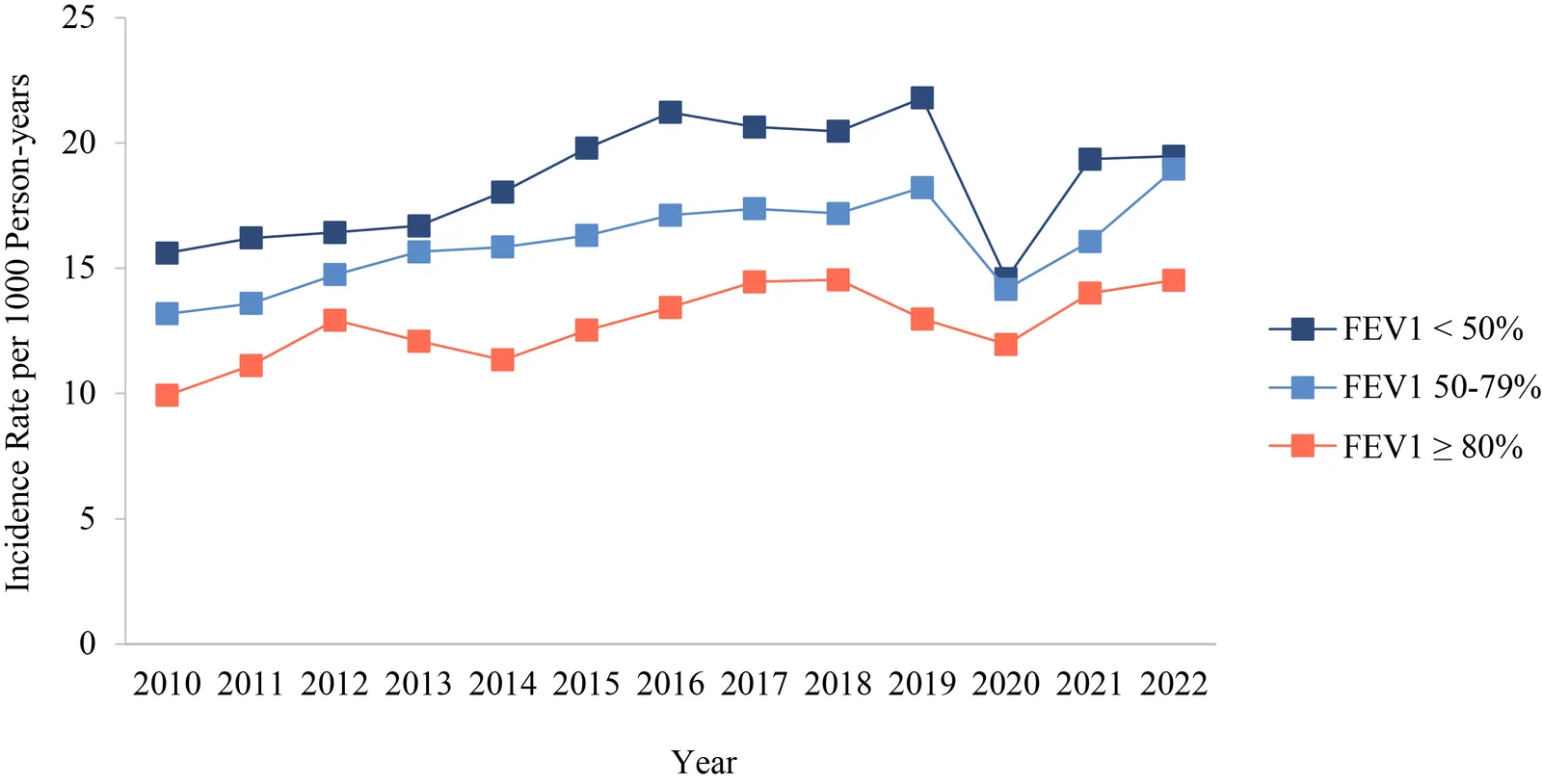
Crude incidence rates of atrial fibrillation per 1000 person-years, stratified by percent predicted forced expiratory volume in 1 s (FEV_1_), in patients with chronic obstructive pulmonary disease in UK primary care from 2010 to 2022. Follow-up intervals with missing FEV_1_ recordings, including 1336 person-years (21.4% of total person-time) and 8783 corresponding events (23.3% of total events), were excluded.

**Fig. 3:**
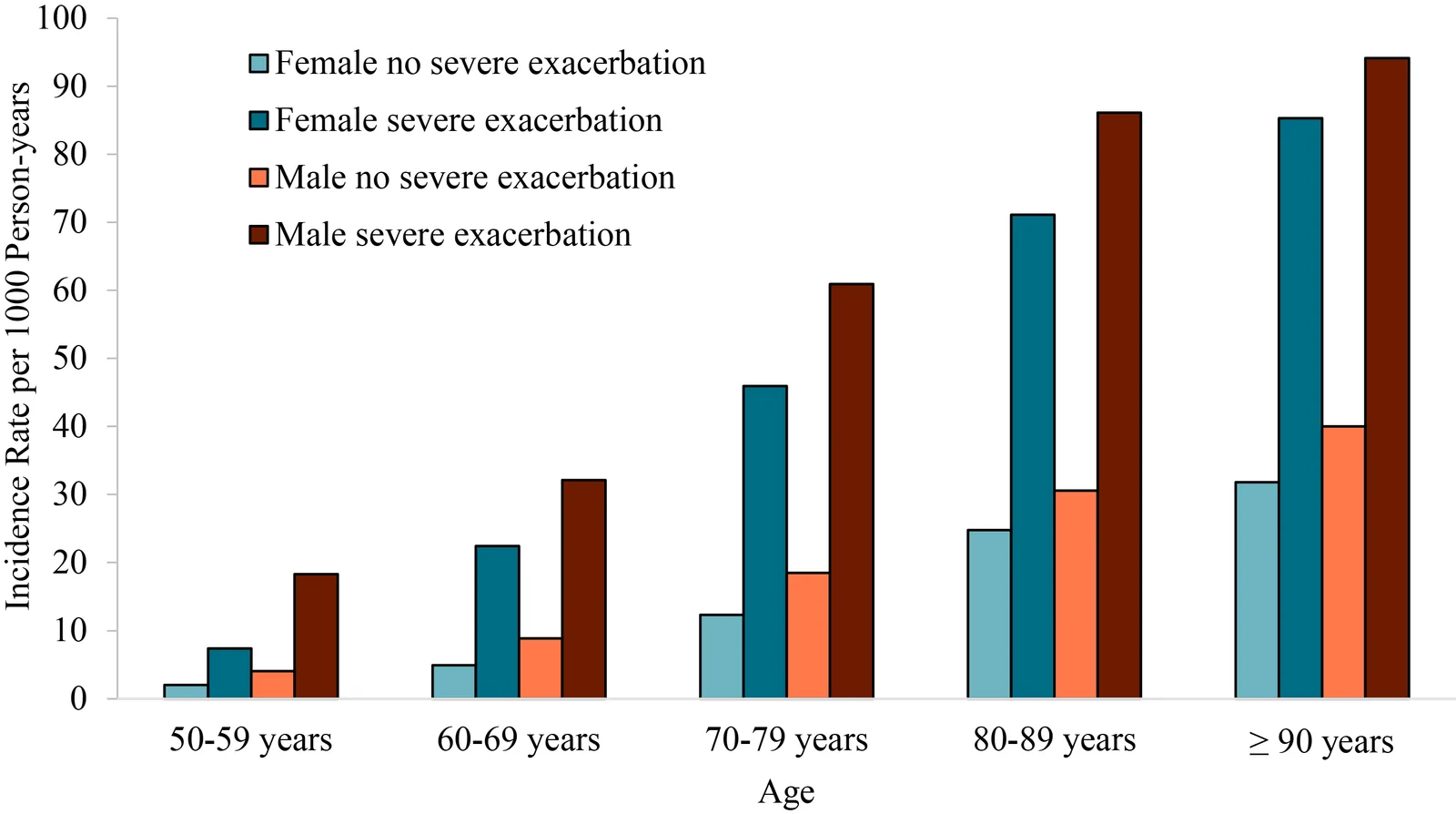
Crude incidence rates of atrial fibrillation per 1000 person-years (2010–2022), stratified by the presence of severe chronic obstructive pulmonary disease (COPD) exacerbation in the prior year, age, and sex, in patients with COPD in UK primary care.

### Trends of OAC initiation among patients with COPD diagnosed with AF

After applying additional exclusion criteria, the sub-cohort of patients with COPD newly diagnosed with AF consisted of 34,851 patients (Figure S13). Among this cohort 23,094 (66.3%) patients were newly prescribed an OAC in the year following AF diagnosis, including 6861 (29.7%) prescribed a VKA and 16,233 (70.3%) prescribed a DOAC. Baseline characteristics of patients newly prescribed an OAC were largely similar to those of patients newly diagnosed with AF; however, cardiovascular disease was more prevalent, and dementia was less prevalent among those prescribed an OAC (Table 1).

Overall, the rate of OAC initiation increased from 10.3 prescriptions per 100 person-months in 2010 to 30.1 per 100 person-months in 2022, with temporary drops in 2011 and 2020 (Figure S14). The annual age- and sex-standardized incidence rates of OAC initiation were similar to the crude rates. The rate of OAC initiation increased over the study period and was 2.9-fold higher in 2022 compared to 2010 (RR: 2.92; 95% CI 2.33, 3.65), with the most prominent increase observed among those aged ≥90 years (RR: 9.30; 95% CI 1.80, 47.94) (Table 3 and Table S3). The rate of VKA initiation decreased from 2014 onward and was 96.0% lower in 2022 than in 2010 (RR: 0.04; 95% CI 0.02, 0.10) (Figure S15 and Table S4). Conversely, the rate of new DOAC use prominently rose from 2012 onwards, with a 64-fold increase from 2012 to 2022 (RR 64.25; 95% CI 33.74, 122.34) (Table S5).

**Table 3:** Incidence rate of oral anticoagulant prescription per 100 person-months in patients with chronic obstructive pulmonary disease and atrial fibrillation in UK primary care, comparing 2022 with 2010, with 95% confidence intervals.

	2010 Rate^a^ (95% CI)	2022 Rate^a^ (95% CI)	RR (95% CI) 2022 versus 2010
Overall	10.33 (8.50, 12.56)	30.16 (27.02, 33.66)	2.92 (2.33, 3.65)
Age (years)			
50–59	17.99 (6.14, 52.73)	13.70 (5.96, 31.50)	0.76 (0.20, 2.97)
60–69	12.65 (8.05, 19.89)	24.01 (17.43, 33.08)	1.90 (1.09, 3.30)
70–79	13.08 (9.97, 17.48)	34.73 (29.29, 41.18)	2.65 (1.90, 3.72)
80–89	8.39 (5.94, 11.85)	32.90 (27.57, 39.27)	3.92 (2.66, 5.78)
≥90	2.11 (0.42, 10.23)	19.59 (12.64, 30.37)	9.30 (1.80, 47.94)
Sex			
Female	8.91 (6.53, 12.15)	31.15 (26.37, 36.80)	3.50 (2.46, 4.97)
Male	11.46 (8.91, 14.74)	29.47 (25.47, 34.11)	2.57 (1.92, 3.44)
Ethnicity^c^			
White	10.51 (8.63, 12.81)	30.10 (26.87, 33.71)	2.86 (2.28, 3.60)
Other^b^	5.96 (1.32, 26.90)	24.31 (13.28, 44.52)	4.08 (0.80, 20.67)
Dyspnea^c^			
no-mild	10.36 (5.86, 18.31)	33.03 (24.16, 45.16)	3.19 (1.66, 6.11)
moderate-severe	10.41 (6.87, 15.77)	28.62 (22.60, 36.25)	2.75 (1.70, 4.43)
Percent predicted FEV1^c^			
≥ 80%	9.95 (3.31, 29.94)	29.17 (15.39, 55.28)	2.93 (0.82, 10.47)
50–<80%	11.32 (6.94, 18.47)	33.03 (22.63, 48.20)	2.92 (1.57, 5.41)
< 50%	10.25 (5.78, 18.18)	36.28 (21.30, 61.80)	3.54 (1.62, 7.74)
Moderate-severe COPD exacerbation			
none	10.27 (6.51, 16.22)	29.71 (23.18, 38.09)	2.89 (1.72, 4.87)
≥1	10.33 (6.66, 16.00)	30.75 (23.81, 39.71)	2.98 (1.79, 4.94)
Severe COPD exacerbation			
none	10.68 (7.51, 15.20)	30.71 (25.25, 37.36)	2.88 (1.92, 4.30)
≥1	8.52 (4.14, 17.52)	26.93 (17.37, 41.75)	3.16 (1.36, 7.36)

Abbreviations: RR, rate ratio; CI, confidence interval; COPD, chronic obstructive pulmonary disease; FEV_1_, forced expiratory volume in 1 s.

a Crude incidence rate per 100 person-months.

b Other includes individuals identifying as Black African, Black Caribbean, any other Black, African, or Caribbean background, Indian, Pakistani, Bangladeshi, Chinese, any other Asian background, Mixed, or any other ethnic group.

c Person-time and corresponding events were excluded for follow-up intervals with missing ethnicity [1.7% (3.7 person-months) of total person-time and 1.0% (7 events) of total events in 2010; 2.6% (6 person-months) of total person time and 3.5% (78 events) of total events in 2022], dyspnea [10.6% (24 person-months) of total person-time and 9.8% (69 events) of total events in 2010; 9.2% (22 person-months) of total person-time and 9.4% (209 events) of total events in 2022], or severity classifications for FEV_1_ [21.6% (49 person-months) of total person-time and 18.2% (128 events) of total events in 2010; 61.7% (150 person-months) of total person-time and 58.1% (1295 events) of total events in 2022].

Throughout the study period, the rate of dabigatran initiation remained low, while apixaban use increased from 2013 onwards, reaching a peak of 208.1 prescriptions per 1000 person-months in 2021 (Fig. 4). The rate of rivaroxaban initiation increased between 2012 and 2016, reaching a peak of 76.5 prescriptions per 1000 person-months in 2016, before steadily declining thereafter. Edoxaban use steadily increased following its introduction in 2016, surpassing rivaroxaban use in 2020 and spiking from 49.4 prescriptions per 1000 person-months in 2021 to 95.4 prescriptions per 1000 person-months in 2022. During the later years (2020–2022), apixaban was the most frequently prescribed OAC (61.6% of all OACs), followed by edoxaban (22.1%), rivaroxaban (13.6%), VKAs (2.1%), and dabigatran (0.6%) (Table S6).

**Fig. 4:**
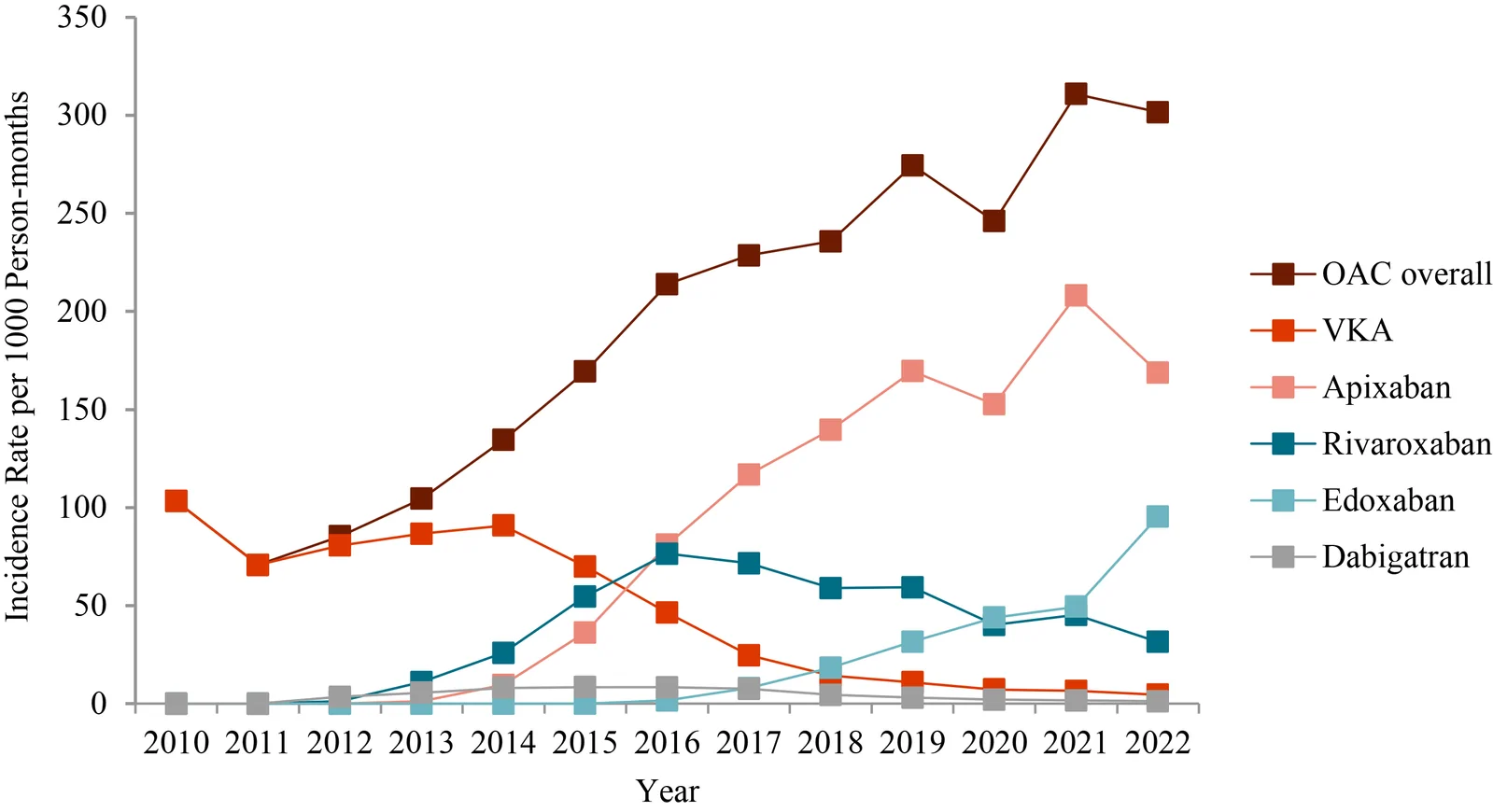
Crude rates of new users of oral anticoagulants (OACs) overall, vitamin K antagonists (VKAs), and individual direct oral anticoagulants, in the year following atrial fibrillation diagnosis per 1000 person-months, in patients with chronic obstructive pulmonary disease in UK primary care from 2010 to 2022.

Overall, DOAC and VKA prescribing was similar between males and females, and this remained consistent across CHA_2_DS_2_-VA score strata (Figures S16-S17). Throughout the study period, patients aged 70–79 years had the highest rates of DOAC and VKA initiation (Figure S18). The proportion of prescriptions attributable to each OAC was consistent across all age groups and in both sexes, apart from VKAs, which were prescribed less frequently among those aged ≥80 years (Figure S19). The proportion of patients prescribed an OAC was slightly lower in females than in males in the oldest and youngest age groups. Those aged 70–79 years had the highest proportion of patients prescribed an OAC, while those aged ≥90 years had the lowest. Across the study period, White patients had higher rates of OAC initiation compared with those of other ethnic backgrounds (Figure S20). OAC prescribing was also similar across geographic region of practice (Figure S21). Patients with more severe dyspnea, lower percent predicted FEV_1_, and a history of a severe COPD exacerbation in the prior year had lower rates of OAC initiation compared to those with milder COPD (Figures S22-S24). Differences were less pronounced between those with and without a moderate or severe COPD exacerbation in the prior year (Figure S25). These patterns were consistent by age and sex (Fig. 5 and Figures S26-S28).

**Fig. 5:**
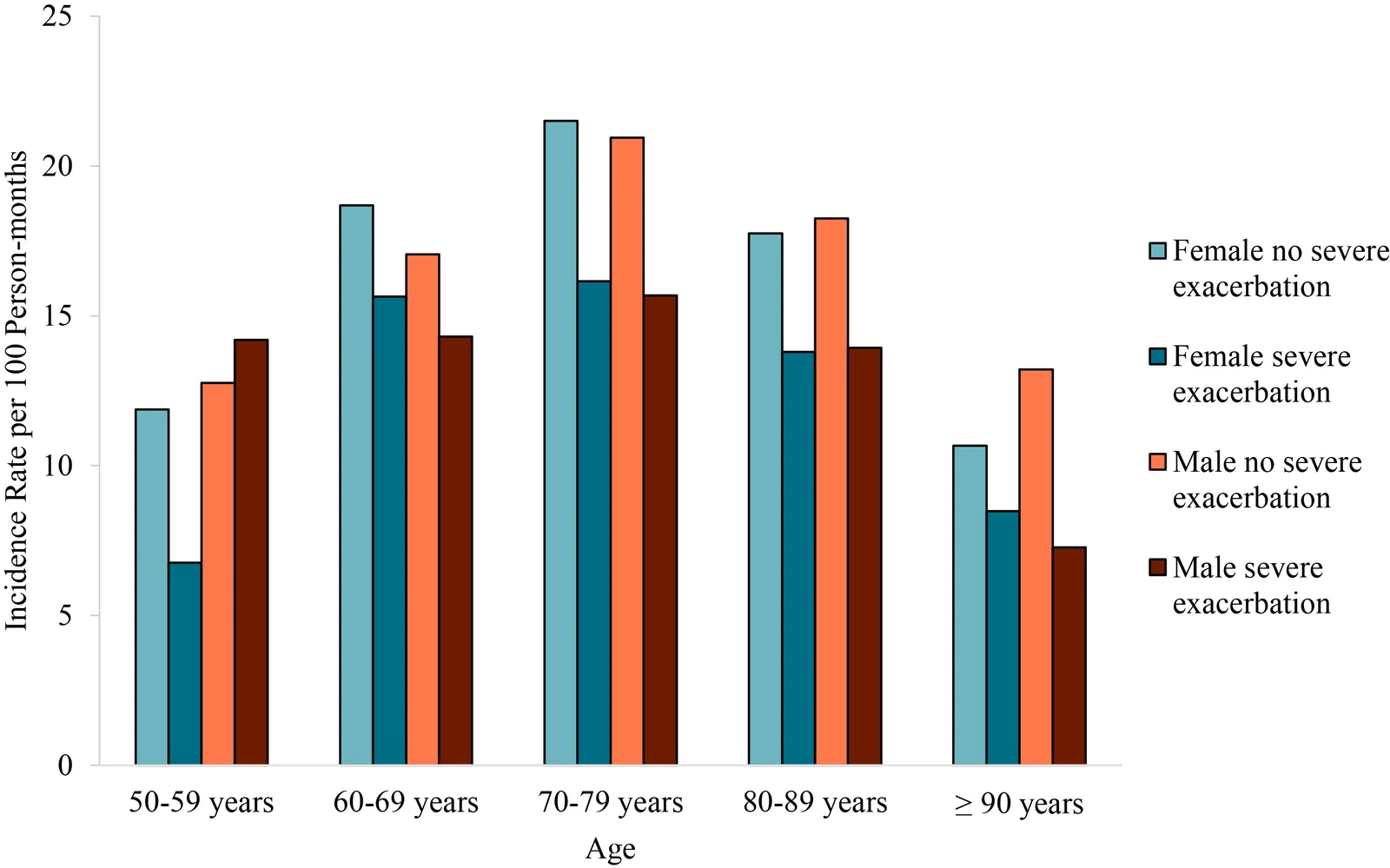
Crude rates of new users of oral anticoagulants in the year following atrial fibrillation diagnosis per 100 person-months (2010–2022), stratified by the presence of severe chronic obstructive pulmonary disease (COPD) exacerbation in the prior year, age, and sex, in patients with COPD in UK primary care.

Between 2016 and 2022, baseline characteristics were largely similar across patients newly prescribed each OAC, with some notable differences (Table S7). Those prescribed a VKA had a higher prevalence of coronary artery disease, congestive heart failure, and chronic kidney disease, but a lower prevalence of dementia compared to those prescribed a DOAC. Overall, those initiated on apixaban had a higher frequency of cardiovascular comorbidities, history of bleeding, and dementia compared to those initiated on other DOACs. Patients prescribed an OAC in the year following AF diagnosis were more likely to be between the ages of 60–89, to have hypertension, history of stroke, diabetes, and obstructive sleep apnea, but less likely to have cardiovascular disease, chronic kidney disease, liver disease, history of bleeding, cancer, dementia, and severe COPD versus those not prescribed an OAC (Table S8). Patients initiated on a DOAC were more likely to be older, to have a history of stroke, dementia, and severe COPD compared to those initiated on a VKA, but less likely to have chronic kidney disease or congestive heart failure (Table S9). These results were consistent in analyses using multiple imputation (data not shown).

The prevalence of beta blocker use in the year following AF diagnosis increased over the study period, from 35.0% (715 patients prescribed) in 2010 to 64.4% (2962 patients prescribed) in 2022, while the use of digitalis glycosides, calcium channel blockers, and antiarrhythmics gradually decreased (Figure S29). Overall, the use of rate- and rhythm-control strategies remained similar in males and females, decreased with age, and was comparable between those with and without a prior COPD exacerbation (Figures S30-S31). The percent of patients treated with LAMA or LABA, alone or in combination in the 3 months prior to AF diagnosis was comparable with the 3 months after AF diagnosis (Figure S32).

## Discussion

In this large population-based cohort study, we found that the incidence of AF gradually increased in patients with COPD in UK primary care between 2010 and 2022. AF incidence was highest among males, older individuals and those with more severe COPD. Among patients newly diagnosed with AF, the rate of OAC initiation nearly tripled over the study period. This increase was driven by a prominent rise in DOAC prescribing from 2012 onwards, alongside a substantial decline in VKA prescribing. OACs were prescribed less frequently to individuals aged ≥90 years, non-White patients, those with severe COPD, and those with chronic kidney disease, liver disease, bleeding, cancer, and dementia.

Prior evidence of temporal trends in the incidence of AF among patients with COPD remains scarce.^8^^,^^30^ A study from Spain showed that the incidence of hospitalization for AF in patients with COPD decreased from 2004 to 2009 followed by an increase from 2009 to 2013, though it did not capture outpatient diagnoses or trends beyond 2013.^8^ Another study from the UK reported an overall increase in the incidence of AF between 1998 and 2019, while the percent of patients with COPD among those newly diagnosed with AF remained relatively stable during this period.^30^ Two additional UK-based cohort studies similarly observed rising incidence rates of AF between 2010 and 2015; thereafter, one reported a declining rate between 2015 and 2018, while the other found stable rates from 2015 to 2019.^31^^,^^32^ Using a well-defined cohort of patients with COPD, we showed a comparable gradual increase in the rate of new AF diagnoses from 2010 to 2022, with a plateau between 2016 and 2019. These similarities suggest that the rising incidence of AF is not unique to patients with COPD and may instead reflect improved detection and increased survival among individuals with risk factors for AF.^33^ We also captured a temporary decline in 2020, likely reflecting reduced access to primary care services during the COVID-19 pandemic.^34^ Consistent with previous studies, we found that the incidence of AF was highest among males, older patients, White individuals, and those with more severe COPD.^5^, ^6^, ^7^^,^^35^^,^^36^ However, unlike prior studies, we incorporated time-varying measures of COPD severity and found that incidence of AF was consistently higher among those with more severe COPD, regardless of the severity marker used.^5^, ^6^, ^7^ Aside from traditional risk factors for AF, our results show that severe COPD plays a distinct role in the occurrence of AF in this population.

While this is the first study to assess temporal OAC prescribing trends in patients with COPD and AF, limited prior cross-sectional data suggested a shift toward DOAC use in this population following their 2011 approval, aligning with our findings.^14^^,^^37^ Patients with prevalent AF and COPD, enrolled in a European registry and managed by cardiologists, were more often prescribed VKAs (78.9% of patients) than DOACs (4.4%) in 2012.^37^ In contrast, a global AF registry covering 2011–2016 reported higher DOAC use (56%) than VKA use (30%) in patients with COPD and newly diagnosed AF, likely reflecting more recent prescribing patterns.^14^ As for individual DOACs, only one study from Taiwan reported their use in patients with COPD and newly diagnosed AF.^38^ However, this study only reported the proportion of patients prescribed each OAC across the entire study period (2002–2015), providing limited insight into DOAC prescribing over time.^38^ In our study of patients with COPD and incident AF, covering 2010–2022, DOAC use prominently increased, surpassing VKA use from 2015 onwards, with apixaban emerging as the most commonly initiated OAC. Notably, the prescribing trends for each OAC observed in our study closely match those previously reported in patients with AF in the UK, though prior studies did not capture new use of individual DOACs beyond 2017.^15^^,^^32^^,^^39^ These similarities suggest that COPD may not have influenced the choice of OAC agent. Nonetheless, further research into the unique clinical considerations of OAC use in patients with both COPD and AF may help guide optimal stroke prevention strategies for this high-risk population.

Despite the overall rise in OAC use following the introduction of DOACs, evidence continues to show underutilization among older adults and high-risk groups, consistent with our findings.^40^^,^^41^ Stroke risk significantly increases with advancing age and AF management guidelines recommend OACs for most individuals with AF aged ≥75 years.^11^ Nonetheless, studies of patients with AF have shown that those aged ≥90 years were less likely to be prescribed an OAC compared to those aged 75–84 years.^40^^,^^41^ Several studies have also shown that females, particularly those aged ≥85 years, have lower rates of OAC initiation than males, potentially related to provider perceptions of frailty and bleeding risk.^40^, ^41^, ^42^ In patients with COPD and AF, we similarly found that females aged ≥90 years had the lowest crude rates of OAC initiation. OAC prescribing was also persistently lower among non-White patients, consistent with findings from studies in patients with AF.^32^^,^^43^ Moreover, OAC initiation was lowest among those with more severe COPD, and this was consistent across all years, ages, sexes, and markers of COPD severity. Although no prior study has assessed OAC use by COPD severity, COPD has been associated with a lower likelihood of OAC treatment in cohort studies of patients with AF.^41^^,^^44^ Our study also showed that patients with a history of some cardiovascular comorbidities, chronic kidney disease, liver disease, bleeding, cancer, or dementia were less likely to be prescribed an OAC compared to patients without these comorbidities. These findings align with prior research, suggesting that concerns about bleeding risk, frailty, polypharmacy, and multimorbidity may contribute to more careful consideration of OAC prescribing in this population.^40^ Additionally, the limited evidence on the safety and efficacy of OACs in patients with both COPD and AF may contribute to physician hesitancy in initiating anticoagulation in this population, particularly in patients with severe COPD.^13^^,^^45^

Collectively, our study highlights a rising incidence of AF, as well as a potential treatment gap among patients with COPD, especially in older patients, those with severe disease, and those with multiple comorbidities. However, the observed trends may not solely reflect inappropriate care as contraindications and patient preferences may also influence prescribing decisions. These findings underscore the importance of a comprehensive integrated care approach for the diagnosis and management of coexisting AF and COPD to optimize treatment and reduce the risk of adverse outcomes. Current AF management guidelines advocate for integrated care approaches, including the AF Better Care pathway, which has been associated with improved outcomes in clinically complex patients.^46^ Studies assessing the cost-effectiveness and benefits of integrated care approaches, as well as routine bidirectional screening for AF and COPD, identified as a priority research area by the American Thoracic Society, may help refine optimal care models for patients with both conditions.^47^ Further research is also warranted to assess the impact of OAC use and other pharmacological therapies on clinical outcomes in this population, particularly among those with high comorbidity burden. Finally, our results may not be generalizable beyond the UK due to differences in underlying population characteristics, risk profiles, access to care, and reimbursement systems.^3^^,^^16^ Future studies assessing trends in the incidence of AF and related treatments in patients with COPD outside of the UK and European context may support region-specific resource allocation and care strategies.

The present study was conducted using the UK's CPRD, which provided a large and representative study population, ensuring an accurate depiction of the long-term trends in the incidence of AF and OAC prescribing for patients with COPD, which have not been previously reported. Moreover, we linked the CPRD data with hospitalization records to define severe COPD exacerbation, which are less subject to misclassification.^18^^,^^48^ A limitation is that the CPRD does not include prescriptions issued by specialists or during hospitalizations, which can lead to underestimating the prescription rates. However, since general practitioners are central to the UK health system, we assume that prescriptions initiated by specialists would be renewed by general practitioners, thus mitigating this risk of misclassification. Secondly, the use of physician diagnoses to identify the cohort may have misclassified some patients with asthma as COPD, though restricting the study population to those aged ≥50 years likely reduced this potential misclassification. Third, per the CPRD-approved protocol, our cohort included only individuals with COPD; thus, we did not directly compare trends with the non-COPD population. However, we provided an indirect comparison with findings from UK-based studies in the Discussion. Fourth, the findings from our ethnicity-stratified analyses should be interpreted cautiously because the small number of patients in non-White ethnic groups required aggregating them into a single category, precluding assessment of heterogeneity between ethnic minority subgroups. Fifth, estimates from the logistic and Cox proportional hazards models may be influenced by missing data. However, results were consistent with analyses using multiple imputation. Finally, patients with missing FEV_1_ or dyspnea recorded in the prior three years did not contribute to stratified analyses for those periods. Nevertheless, by treating these variables as time-varying and incorporating three distinct markers of COPD severity, we maximized available data, providing a comprehensive characterization of disease severity over time.

In summary, from 2010 to 2022, both the incidence of AF and OAC initiation increased in patients with COPD in UK primary care. AF incidence was highest among older individuals and those with more severe COPD. However, OAC use may remain less than optimal among these sub-groups, revealing a potential treatment gap in those at greatest risk. Further research focused on the complex clinical interplay between COPD and AF, including its impact on anticoagulant treatment decisions and outcomes, will be essential to inform optimal stroke prevention strategies in this high-risk population.

## Contributors

BM, PE, SS, and CR designed the study. BM and SB conducted the statistical analysis. BM, SS, and CR contributed to the literature search and acquired and interpreted the data. BM, SB, SS and CR accessed and verified the underlying data. BM wrote the first draft of the manuscript. BM, PE, SS, and CR reviewed and edited the manuscript. SS and CR provided supervision. All authors approved the final manuscript and had final responsibility for the decision to submit it.

## Data sharing statement

This study is based in part on data from the Clinical Practice Research Datalink obtained under license from the UK Medicines and Healthcare products Regulatory Agency. The data are provided by patients and collected by the UK National Health Service as part of their care and support. The interpretation and conclusions contained in this study are those of the authors alone. Because electronic health records are classified as “sensitive data” by the UK Data Protection Act, information governance restrictions (to protect patient confidentiality) prevent data sharing via public deposition. Data are available with approval through the individual constituent entities controlling access to the data. Specifically, the primary care data can be requested via application to the Clinical Practice Research Datalink (https://www.cprd.com).

## Declaration of interests

In the last three years, S Suissa attended advisory committee meetings for Atara, Novartis and Panalgo, and received speaking fees from Amgen, CSL Behring, and Covis Pharma. No other conflicts of interest to declare.
