# Supplementary material for: Trends in the incidence of atrial fibrillation and the prescription of oral anticoagulants in adults with chronic obstructive pulmonary disease in UK primary care (2010–2022): a cohort study
Source: eClinicalMedicine. 2026 Sep 28;100:104245. doi: 10.1016/j.eclinm.2026.104245 (PMC13651490; doi:10.1016/j.eclinm.2026.104245)
Supplement: Supplementary file 1 [file mmc1.docx]

**SUPPLEMENTARY MATERIALS**

**Trends in the incidence of atrial fibrillation and the prescription of oral anticoagulants in adults with chronic obstructive pulmonary disease in UK Primary Care (2010-2022): a cohort study**

Brianna Murray, MSc, Sarah Beradid, MSc, Pierre Ernst, MD, MSc, Samy Suissa, PhD, Christel Renoux, MD, PhD

[Table S1. Incidence rate ratios of atrial fibrillation in patients with chronic obstructive pulmonary disease in UK primary care, comparing the rate for each calendar year to that in 2010 and the preceding year, adjusted for age and sex, with 95% confidence intervals	1](#_Toc228974050)

[Table S2. Hazard ratios and 95% confidence intervals for the association between patient characteristics and the incidence of atrial fibrillation in patients with chronic obstructive pulmonary disease in UK primary care from 2010 to 2022, conditioned on calendar year	2](#_Toc228974051)

[Table S3. Incidence rate ratios of oral anticoagulant prescription in patients with chronic obstructive pulmonary disease in UK primary care, comparing the rate (measured in the year following atrial fibrillation diagnosis) for each calendar year to that in 2010 and the preceding year, adjusted for age and sex, with 95% confidence intervals	4](#_Toc228974052)

[Table S4. Incidence rate ratios of vitamin K antagonist prescription in patients with chronic obstructive pulmonary disease in UK primary care, comparing the rate (measured in the year following atrial fibrillation diagnosis) for each calendar year to that in 2010 and the preceding year, adjusted for age and sex, with 95% confidence intervals	5](#_Toc228974053)

[Table S5. Incidence rate ratios of direct oral anticoagulant prescription in patients with chronic obstructive pulmonary disease in UK primary care, comparing the rate (measured in the year following atrial fibrillation diagnosis) for each calendar year to that in 2012 and the preceding year, adjusted for age and sex, with 95% confidence intervals	6](#_Toc228974054)

[Table S6. Percent of prescriptions in the year following atrial fibrillation diagnosis in patients with chronic obstructive pulmonary disease in UK primary care attributable to each oral anticoagulant in each calendar year	7](#_Toc228974055)

[Table S7. Baseline characteristics of patients with chronic obstructive pulmonary disease newly prescribed an oral anticoagulant in the year following atrial fibrillation diagnosis between 2016 and 2022	8](#_Toc228974056)

[Table S8. Hazard ratios and 95% confidence intervals for the association between patient characteristics and the initiation of an oral anticoagulant in the year following atrial fibrillation diagnosis in patients with chronic obstructive pulmonary disease in UK primary care from 2010 to 2022, conditioned on calendar year	11](#_Toc228974057)

[Table S9. Odds ratios and 95% confidence intervals for the association between patient characteristics and the initiation of direct oral anticoagulants compared to the initiation of vitamin K antagonists in the year following atrial fibrillation diagnosis in patients with chronic obstructive pulmonary disease in UK primary care from 2010 to 2022, conditioned on calendar year	13](#_Toc228974058)

[Figure S1. Flowchart of patient selection in study cohort of patients with chronic obstructive pulmonary disease in UK primary care (2010-2022)	15](#_Toc228974059)

[Figure S2. Crude and standardized (to the age and sex distribution of the study cohort in the year 2022) incidence rates of atrial fibrillation per 1,000 person-years, in patients with chronic obstructive pulmonary disease in UK primary care from 2010 to 2022	16](#_Toc228974060)

[Figure S3. Crude incidence rates of atrial fibrillation per 1,000 person-years overall and stratified by sex, in patients with chronic obstructive pulmonary disease in UK primary care from 2010 to 2022	17](#_Toc228974061)

[Figure S4. Crude incidence rates of atrial fibrillation per 1,000 person-years, stratified by age, in patients with chronic obstructive pulmonary disease in UK primary care from 2010 to 2022	18](#_Toc228974062)

[Figure S5. Crude incidence rates of atrial fibrillation per 1,000 person-years, stratified by ethnicity, in patients with chronic obstructive pulmonary disease in UK primary care from 2010 to 2022	19](#_Toc228974063)

[Figure S6. Crude incidence rates of atrial fibrillation per 1,000 person-years, stratified by geographic region of practice, in patients with chronic obstructive pulmonary disease in UK primary care from 2010 to 2022	20](#_Toc228974064)

[Figure S7. Crude incidence rates of atrial fibrillation per 1,000 person-years, stratified by level of dyspnea, in patients with chronic obstructive pulmonary disease in UK primary care from 2010 to 2022	21](#_Toc228974065)

[Figure S8. Crude incidence rates of atrial fibrillation per 1,000 person-years, stratified by the presence of severe chronic obstructive pulmonary disease (COPD) exacerbation in the prior year, in patients with COPD in UK primary care from 2010 to 2022	22](#_Toc228974066)

[Figure S9. Crude incidence rates of atrial fibrillation per 1,000 person-years, stratified by the presence of moderate or severe chronic obstructive pulmonary disease (COPD) exacerbation in the prior year, in patients with COPD in UK primary care from 2010 to 2022	23](#_Toc228974067)

[Figure S10. Crude incidence rates of atrial fibrillation per 1,000 person-years (2010-2022), stratified by level of dyspnea, age, and sex, in patients with chronic obstructive pulmonary disease in UK primary care	24](#_Toc228974068)

[Figure S11. Crude incidence rates of atrial fibrillation per 1,000 person-years (2010-2022), stratified by percent predicted forced expiratory volume in one second (FEV_1_), age, and sex, in patients with chronic obstructive pulmonary disease in UK primary care	25](#_Toc228974069)

[Figure S12. Crude incidence rates of atrial fibrillation per 1,000 person-years (2010-2022), stratified by the presence of moderate or severe chronic obstructive pulmonary disease (COPD) exacerbation in the prior year, age, and sex, in patients with COPD in UK primary care	26](#_Toc228974070)

[Figure S13. Flowchart of patient selection in sub-cohort of patients with chronic obstructive pulmonary disease and newly diagnosed atrial fibrillation in UK primary care (2010-2022)	27](#_Toc228974071)

[Figure S14. Crude and standardized (to the age and sex distribution of the study cohort in the year 2022) rates of new users of oral anticoagulants in the year following atrial fibrillation diagnosis per 100 person-months, in patients with chronic obstructive pulmonary disease in UK primary care from 2010 to 2022	28](#_Toc228974073)

[Figure S15. Crude rates of new users of oral anticoagulants (OACs) in the year following atrial fibrillation diagnosis per 100 person-months overall, and stratified by direct oral anticoagulants (DOACs) and vitamin K antagonists (VKAs), in patients with chronic obstructive pulmonary disease in UK primary care from 2010 to 2022	29](#_Toc228974074)

[Figure S16. Crude rates of new users of vitamin K antagonists (VKAs) and direct oral anticoagulants (DOACs) in the year following atrial fibrillation diagnosis per 100 person-months, stratified by sex, in patients with chronic obstructive pulmonary disease in UK primary care from 2010 to 2022	30](#_Toc228974075)

[Figure S17. Crude rates of new users of oral anticoagulants in the year following atrial fibrillation diagnosis per 100 person-months, stratified by sex and CHA_2_DS_2_-VA score, in patients with chronic obstructive pulmonary disease in UK primary care from 2010 to 2022	31](#_Toc228974076)

[Figure S18. Crude rates of new users of vitamin K antagonists (VKAs) and direct oral anticoagulants (DOACs) in the year following atrial fibrillation diagnosis per 100 person-months, stratified by age, in patients with chronic obstructive pulmonary disease in UK primary care from 2010 to 2022	32](#_Toc228974077)

[Figure S19. Percent of patients newly prescribed an oral anticoagulant in the year following atrial fibrillation diagnosis, stratified by age and sex, in patients with chronic obstructive pulmonary disease in UK primary care 2010-2022	33](#_Toc228974078)

[Figure S20. Crude rates of new users of oral anticoagulants in the year following atrial fibrillation diagnosis per 100 person-months, stratified by ethnicity, in patients with chronic obstructive pulmonary disease in UK primary care from 2010 to 2022	34](#_Toc228974079)

[Figure S21. Crude rates of new users of oral anticoagulants in the year following atrial fibrillation diagnosis per 100 person-months, stratified by geographic region of practice, in patients with chronic obstructive pulmonary disease in UK primary care from 2010 to 2022	35](#_Toc228974080)

[Figure S22. Crude rates of new users of vitamin K antagonists (VKAs) and direct oral anticoagulants (DOACs) in the year following atrial fibrillation diagnosis per 100 person-months, stratified by level of dyspnea, in patients with chronic obstructive pulmonary disease in UK primary care from 2010 to 2022	36](#_Toc228974081)

[Figure S23. Crude rates of new users of vitamin K antagonists (VKAs) and direct oral anticoagulants (DOACs) in the year following atrial fibrillation diagnosis per 100 person-months, stratified by percent predicted forced expiratory volume in one second (FEV_1_), in patients with chronic obstructive pulmonary disease in UK primary care from 2010 to 2022	37](#_Toc228974082)

[Figure S24. Crude rates of new users of vitamin K antagonists (VKAs) and direct oral anticoagulants (DOACs) in the year following atrial fibrillation diagnosis per 100 person-months, stratified by the presence of severe chronic obstructive pulmonary disease (COPD) exacerbation in the prior year, in patients with COPD in UK primary care from 2010 to 2022	38](#_Toc228974083)

[Figure S25. Crude rates of new users of vitamin K antagonists (VKAs) and direct oral anticoagulants (DOACs) in the year following atrial fibrillation diagnosis per 100 person-months, stratified by the presence of moderate or severe chronic obstructive pulmonary disease (COPD) exacerbation in the prior year, in patients with COPD in UK primary care from 2010 to 2022	39](#_Toc228974084)

[Figure S26. Crude rates of new users of oral anticoagulants in the year following atrial fibrillation diagnosis per 100 person-months (2010-2022), stratified by level of dyspnea, age, and sex, in patients with chronic obstructive pulmonary disease in UK primary care	40](#_Toc228974085)

[Figure S27. Crude rates of new users of oral anticoagulants in the year following atrial fibrillation diagnosis per 100 person-months (2010-2022), stratified by percent predicted forced expiratory volume in one second (FEV_1_), age, and sex, in patients with chronic obstructive pulmonary disease in UK primary care	41](#_Toc228974086)

[Figure S28. Crude rates of new users of oral anticoagulants in the year following atrial fibrillation diagnosis per 100 person-months (2010-2022), stratified by the presence of moderate or severe chronic obstructive pulmonary disease (COPD) exacerbation in the prior year, age, and sex in patients with COPD in UK primary care	42](#_Toc228974087)

[Figure S29. Prevalence of beta blocker, digitalis glycosides, calcium channel blocker and antiarrhythmic drug therapy use, and ablation procedures for atrial fibrillation, in the year following atrial fibrillation diagnosis, stratified by sex, in patients with chronic obstructive pulmonary disease in UK primary care from 2010 to 2022	43](#_Toc228974088)

[Figure S30. Prevalence of beta blocker, digitalis glycosides, calcium channel blocker, and antiarrhythmic drug therapy in the year following atrial fibrillation diagnosis, stratified by age, in patients with chronic obstructive pulmonary disease and in UK primary care from 2010 to 2022	44](#_Toc228974089)

[Figure S31. Prevalence of beta blocker, digoxin, calcium channel blocker and antiarrhythmic drug therapy use, and ablation procedures for atrial fibrillation, in the year following atrial fibrillation diagnosis, stratified by chronic obstructive pulmonary disease (COPD) exacerbation in the prior year, in patients with COPD in UK primary care from 2010 to 2022	45](#_Toc228974090)

[Figure S32. Percent of patients prescribed long-acting muscarinic antagonists or long-acting beta agonists alone or in combination, in the 3 months prior to and the three months after atrial fibrillation diagnosis in patients with chronic obstructive pulmonary disease in UK primary care from 2010 to 2022	46](#_Toc228974091)

Table S1. Incidence rate ratios of atrial fibrillation in patients with chronic obstructive pulmonary disease in UK primary care, comparing the rate for each calendar year to that in 2010 and the preceding year, adjusted for age and sex, with 95% confidence intervals

| **Year** | **Rate* (95% CI)** | **RR** compared with 2010**  **(95% CI)** | **RR** compared with preceding**  **year (95% CI)** |
| --- | --- | --- | --- |
| 2010 | 13.82 (11.92, 16.02) | 1.00 (reference) | 1.00 (reference) |
| 2011 | 14.50 (12.60, 16.70) | 1.05 (0.86, 1.29) | 1.05 (0.86, 1.29) |
| 2012 | 15.40 (13.47, 17.60) | 1.11 (0.91, 1.36) | 1.06 (0.87, 1.29) |
| 2013 | 15.72 (13.80, 17.91) | 1.14 (0.94, 1.39) | 1.02 (0.85, 1.24) |
| 2014 | 15.99 (14.08, 18.17) | 1.16 (0.95, 1.41) | 1.02 (0.85, 1.22) |
| 2015 | 17.12 (15.16, 19.34) | 1.24 (1.03, 1.50) | 1.07 (0.90, 1.28) |
| 2016 | 17.57 (15.61, 19.77) | 1.27 (1.05, 1.54) | 1.02 (0.86, 1.21) |
| 2017 | 18.01 (16.05, 20.21) | 1.30 (1.08, 1.57) | 1.02 (0.87, 1.21) |
| 2018 | 17.81 (15.89, 19.97) | 1.29 (1.07, 1.55) | 0.99 (0.84, 1.16) |
| 2019 | 17.97 (16.04, 20.13) | 1.29 (1.07, 1.56) | 1.00 (0.85, 1.18) |
| 2020 | 14.08 (12.38, 16.02) | 1.01 (0.83, 1.23) | 0.78 (0.66, 0.93) |
| 2021 | 16.87 (14.95, 19.03) | 1.19 (0.99, 1.44) | 1.18 (0.99, 1.41) |
| 2022 | 18.34 (16.30, 20.64) | 1.29 (1.07, 1.56) | 1.08 (0.91, 1.28) |

*****Crude incidence rate per 1,000 person-years.

******Adjusted for age and sex.

**Abbreviations**: RR, rate ratio; CI, confidence interval.

Table S2. Hazard ratios and 95% confidence intervals for the association between patient characteristics and the incidence of atrial fibrillation in patients with chronic obstructive pulmonary disease in UK primary care from 2010 to 2022, conditioned on calendar year

|  | **Diagnosed with AF^a^,**  **n =37,645** | **No AF^a^,**  **n = 407,023** | **Crude HR** | **Adjusted* HR (95% CI)** |  |
| --- | --- | --- | --- | --- | --- |
| **Age (years)** |  |  |  |  |  |
| 50-59 | 1966 (5.2) | 69,083 (17.0) | 1.00 (ref.) | 1.00 (ref.) |  |
| 60-69 | 9821 (26.1) | 136,397 (33.5) | 2.37 | 2.16 (2.06, 2.27) |  |
| 70-79 | 16,076 (42.7) | 126,349 (31.0) | 5.28 | 4.14 (3.95, 4.35) |  |
| 80-89 | 8861 (23.5) | 65,070 (16.0) | 9.31 | 6.17 (5.85, 6.50) |  |
| ≥90 | 921 (2.5) | 10,124 (2.5) | 11.64 | 7.58 (6.97, 8.24) |  |
| **Sex** |  |  |  |  |  |
| Female | 15,861 (42.1) | 198,497 (48.8) | 1.00 (ref.) | 1.00 (ref.) |  |
| Male | 21,784 (57.9) | 208,526 (51.2) | 1.34 | 1.38 (1.35, 1.41) |  |
| **Ethnicity** |  |  |  |  |  |
| White | 36,028 (95.7) | 367,040 (90.2) | 1.00 (ref.) | 1.00 (ref.) |  |
| Other^b^ | 907 (2.4) | 15,849 (3.9) | 0.61 | 0.56 (0.52, 0.59) |  |
| Missing**†** | 710 (1.9) | 24,134 (5.9) | 0.33 | 0.46 (0.43, 0.50) |  |
| **Body mass index** |  |  |  |  |  |
| < 30 kg/m2 | 22,003 (58.4) | 264,834 (65.1) | 1.00 (ref,) | 1.00 (ref.) |  |
| ≥ 30 kg/m2 | 12,947 (34.4) | 104,654 (25.7) | 1.39 | 1.36 (1.33, 1.39) |  |
| Missing**†** | 2695 (7.2) | 37,535 (9.2) | 1.00 | 1.09 (1.04, 1.14) |  |
| **Smoking status** |  |  |  |  |  |
| Non-smoker | 5266 (14.0) | 46,572 (11.4) | 1.00 (ref.) | 1.00 (ref.) |  |
| Ex smoker | 15,642 (41.6) | 142,290 (41.6) | 1.01 | 1.00 (0.97, 1.03) |  |
| Smoker | 16,447 (43.7) | 213,934 (43.7) | 0.70 | 0.97 (0.94, 1.00) |  |
| Missing**†** | 290 (0.8) | 4227 (1.0) | 1.01 | 0.97 (0.85, 1.09) |  |
| Alcohol abuse | 2617 (7.0) | 37,003 (9.1) | 0.97 | 1.17 (1.13, 1.22) |  |
| **Comorbidities** |  |  |  |  |  |
| Congestive heart failure | 4675 (12.4) | 33,316 (8.2) | 2.18 | 1.49 (1.44, 1.54) |  |
| Hypertension | 27,881 (74.1) | 241,573 (59.4) | 2.50 | 1.43 (1.39, 1.47) |  |
| Myocardial infarction | 5104 (13.6) | 40,252 (9.9) | 1.79 | 1.10 (1.06, 1.13) |  |
| Peripheral arterial disease | 6104 (16.2) | 52,902 (13.0) | 1.70 | 1.17 (1.14, 1.21) |  |
| Stroke or TIA | 3793 (10.1) | 35,811 (8.8) | 1.51 | 1.06 (1.03, 1.10) |  |
| Systemic embolism | 189 (0.5) | 2120 (0.5) | 1.28 | 0.91 (0.78, 1.05) |  |
| Diabetes | 8307 (22.1) | 81,822 (20.1) | 1.36 | 0.98 (0.95, 1.00) |  |
| Chronic kidney disease | 8467 (22.5) | 68,747 (16.9) | 1.75 | 1.04 (1.01, 1.06) |  |
| Liver disease | 1416 (3.8) | 21,939 (5.4) | 0.97 | 1.02 (0.97, 1.08) |  |
| History of bleeding | 13,582 (36.1) | 150,654 (37.0) | 1.09 | 1.02 (1.00, 1.05) |  |
| Cancer^c^ | 6139 (16.3) | 66,766 (16.4) | 1.27 | 1.03 (1.00, 1.06) |  |
| Dementia | 972 (2.6) | 19,304 (4.7) | 1.39 | 0.76 (0.71, 0.81) |  |
| Obstructive sleep apnea | 1103 (2.9) | 10,346 (2.5) | 1.42 | 1.21 (1.14, 1.29) |  |
| **Level of dyspnea**** |  |  |  |  |  |
| No-mild | 13,488 (35.8) | 142,710 (35.1) | 1.00 (ref.) | 1.00 (ref.) |  |
| Moderate-severe | 14,549 (38.6) | 145,133 (35.7) | 1.41 | 1.14 (1.11, 1.17) |  |
| Missing**†** | 9608 (25.5) | 119,180 (29.3) | 1.07 | 1.05 (1.02, 1.08) |  |
| **Percent predicted FEV_1_**** | | | | | |
| FEV_1_ ≥80% | 5099 (13.5) | 57,833 (14.2) | 1.00 (ref.) | 1.00 (ref.) |  |
| FEV_1_ 50 - <80% | 16,369 (43.5) | 152,886 (37.6) | 1.16 | 1.14 (1.11, 1.18) |  |
| FEV_1_ <50% | 7637 (20.3) | 76,793 (18.9) | 1.27 | 1.22 (1.18, 1.27) |  |
| Missing**†** | 8540 (22.7) | 119,511 (29.4) | 1.24 | 1.17 (1.13, 1.22) |  |
| **Prednisolone prescription***** | 12,100 (32.1) | 128,315 (31.5) | 1.10 | 1.07 (1.05, 1.09) |  |
| **Number of COPD hospitalizations***** | | | | | |
| 0 | 35,937 (95.5) | 385,514 (86.7) | 1.00 (ref.) | 1.00 (ref.) |  |
| 1 | 1450 (3.9) | 17,548 (4.0) | 1.34 | 1.12 (1.06, 1.18) |  |
| ≥2 | 258 (0.7) | 3961 (0.9) | 1.44 | 1.11 (0.98, 1.26) |  |

Age was measured at the time of cohort entry, while all other covariates were measured at any time prior, unless otherwise specified.

**^a^**Values expressed as n (%).

**^b^**Other includes individuals identifying as Black African, Black Caribbean, any other Black, African, or Caribbean background, Indian, Pakistani, Bangladeshi, Chinese, any other Asian background, Mixed, or any other ethnic group.

**^c^**Cancer includes all cancers except non-melanoma skin cancer.

**†**Patients with missing ethnicity, body mass index, smoking status, dyspnea, and percent predicted FEV_1_ recordings, were retained by including a separate category for missing values in the Cox proportional hazards regression models.

*****Adjusted for all variables listed in the table.

******Most recent measure in the prior 3 years.

*******Measured in the year before cohort entry.

**Abbreviations**: HR, hazard ratio; CI, confidence interval; AF, atrial fibrillation; COPD, chronic obstructive pulmonary disease; TIA, transient ischaemic attack; FEV_1_, percent predicted forced expiratory volume in one second; ref., reference.

Table S3. Incidence rate ratios of oral anticoagulant prescription in patients with chronic obstructive pulmonary disease in UK primary care, comparing the rate (measured in the year following atrial fibrillation diagnosis) for each calendar year to that in 2010 and the preceding year, adjusted for age and sex, with 95% confidence intervals

| Year | Rate* (95% CI) | RR** compared with 2010  (95% CI) | RR** compared with preceding  year (95% CI) |
| --- | --- | --- | --- |
| 2010 | 10.33 (8.50, 12.56) | 1.00 (reference) | 1.00 (reference) |
| 2011 | 7.07 (5.98, 8.37) | 0.68 (0.53, 0.89) | 0.68 (0.53, 0.89) |
| 2012 | 8.53 (7.33, 9.93) | 0.83 (0.65, 1.06) | 1.21 (0.96, 1.52) |
| 2013 | 10.45 (9.09, 12.01) | 1.01 (0.80, 1.29) | 1.23 (1.00, 1.51) |
| 2014 | 13.44 (11.81, 15.31) | 1.30 (1.03, 1.64) | 1.28 (1.06, 1.55) |
| 2015 | 16.93 (15.05, 19.05) | 1.64 (1.31, 2.06) | 1.26 (1.06, 1.50) |
| 2016 | 21.39 (19.12, 23.92) | 2.07 (1.65, 2.59) | 1.26 (1.07, 1.48) |
| 2017 | 22.85 (20.48, 25.50) | 2.21 (1.76, 2.76) | 1.07 (0.91, 1.25) |
| 2018 | 23.57 (21.16, 26.26) | 2.28 (1.83, 2.85) | 1.03 (0.89, 1.21) |
| 2019 | 27.44 (24.71, 30.47) | 2.65 (2.13, 3.31) | 1.16 (1.00, 1.35) |
| 2020 | 24.60 (21.79, 27.77) | 2.38 (1.89, 2.99) | 0.90 (0.76, 1.05) |
| 2021 | 31.08 (27.79, 34.75) | 3.01 (2.41, 3.77) | 1.27 (1.07, 1.49) |
| 2022 | 30.16 (27.02, 33.66) | 2.92 (2.33, 3.65) | 0.97 (0.83, 1.13) |

*****Crude incidence rate per 100 person-months.

******Adjusted for age and sex.

**Abbreviations**: RR, rate ratio; CI, confidence interval.

Table S4. Incidence rate ratios of vitamin K antagonist prescription in patients with chronic obstructive pulmonary disease in UK primary care, comparing the rate (measured in the year following atrial fibrillation diagnosis) for each calendar year to that in 2010 and the preceding year, adjusted for age and sex, with 95% confidence intervals

| **Year** | **Rate* (95% CI)** | **RR** compared with 2010**  **(95% CI)** | **RR** compared with preceding**  **year (95% CI)** |
| --- | --- | --- | --- |
| 2010 | 10.33 (8.63, 12.37) | 1.00 (reference) | 1.00 (reference) |
| 2011 | 7.07 (6.06, 8.26) | 0.68 (0.54, 0.87) | 0.68 (0.54, 0.87) |
| 2012 | 8.07 (6.98, 9.32) | 0.78 (0.62, 0.99) | 1.15 (0.93, 1.41) |
| 2013 | 8.66 (7.52, 9.97) | 0.84 (0.67, 1.06) | 1.08 (0.88, 1.32) |
| 2014 | 9.09 (7.86, 10.51) | 0.88 (0.70, 1.10) | 1.04 (0.85, 1.27) |
| 2015 | 6.99 (5.90, 8.28) | 0.68 (0.53, 0.86) | 0.77 (0.62, 0.96) |
| 2016 | 4.64 (3.72, 5.79) | 0.44 (0.33, 0.59) | 0.66 (0.50, 0.87) |
| 2017 | 2.46 (1.81, 3.35) | 0.24 (0.17, 0.34) | 0.53 (0.36. 0.77) |
| 2018 | 1.44 (0.96, 2.16) | 0.14 (0.09, 0.22) | 0.59 (0.36, 0.98) |
| 2019 | 1.09 (0.67, 1.77) | 0.10 (0.06, 0.18) | 0.75 (0.40, 1.41) |
| 2020 | 0.73 (0.38, 1.39) | 0.07 (0.04, 0.14) | 0.66 (0.30, 1.49) |
| 2021 | 0.65 (0.32, 1.32) | 0.06 (0.03, 0.13) | 0.90 (0.34, 2.36) |
| 2022 | 0.46 (0.20, 1.05) | 0.04 (0.02, 0.10) | 0.71 (0.24, 2.09) |

*****Crude incidence rate per 100 person-months.

******Adjusted for age and sex.

**Abbreviations**: RR, rate ratio; CI, confidence interval.

Table S5. Incidence rate ratios of direct oral anticoagulant prescription in patients with chronic obstructive pulmonary disease in UK primary care, comparing the rate (measured in the year following atrial fibrillation diagnosis) for each calendar year to that in 2012 and the preceding year, adjusted for age and sex, with 95% confidence intervals

| **Year** | **Rate* (95% CI)** | **RR** compared with 2012**  **(95% CI)** | **RR** compared with preceding**  **year (95% CI)** |
| --- | --- | --- | --- |
| 2012 | 0.46 (0.25, 0.87) | 1.00 (reference) | 1.00 (reference) |
| 2013 | 1.79 (1.29, 2.48) | 3.86 (1.89, 7.88) | 3.86 (1.89, 7.88) |
| 2014 | 4.36 (3.49, 5.44) | 9.41 (4.80, 18.44) | 2.44 (1.64, 3.62) |
| 2015 | 9.94 (8.56, 11.54) | 21.51 (11.20, 41.31) | 2.29 (1.75, 2.99) |
| 2016 | 16.75 (14.81, 18.93) | 36.20 (18.96, 69.11) | 1.68 (1.39, 2.04) |
| 2017 | 20.39 (18.21, 22.82) | 44.04 (23.11, 83.95) | 1.22 (1.03, 1.44) |
| 2018 | 22.13 (19.86, 24.66) | 47.85 (25.12, 91.13) | 1.09 (0.93, 1.27) |
| 2019 | 26.35 (23.75, 29.24) | 56.97 (29.93, 108.42) | 1.19 (1.02, 1.38) |
| 2020 | 23.87 (21.18, 26.90) | 51.61 (27.04, 98.49) | 0.91 (0.77, 1.06) |
| 2021 | 30.43 (27.27, 33.96) | 65.86 (34.57, 125.46) | 1.28 (1.08, 1.50) |
| 2022 | 29.70 (26.67, 33.07) | 64.25 (33.74, 122.34) | 0.98 (0.84, 1.14) |

*****Crude incidence rate per 100 person-months.

******Adjusted for age and sex.

**Abbreviations**: RR, rate ratio; CI, confidence interval.

Table S6. Percent of prescriptions in the year following atrial fibrillation diagnosis in patients with chronic obstructive pulmonary disease in UK primary care attributable to each oral anticoagulant in each calendar year

| Year | VKA | DOAC* | Dabigatran | Rivaroxaban | Apixaban | Edoxaban |
| --- | --- | --- | --- | --- | --- | --- |
| 2010 | 100.0 | 0.0 | 0.0 | 0.0 | 0.0 | 0.0 |
| 2011 | 100.0 | 0.0 | 0.0 | 0.0 | 0.0 | 0.0 |
| 2012 | 94.6 | 5.4 | 4.0 | 1.4 | 0.0 | 0.0 |
| 2013 | 82.9 | 17.1 | 5.4 | 10.5 | 1.2 | 0.0 |
| 2014 | 67.6 | 32.4 | 6.0 | 19.2 | 7.1 | 0.0 |
| 2015 | 41.3 | 58.7 | 5.0 | 32.3 | 21.4 | 0.0 |
| 2016 | 21.7 | 78.3 | 3.9 | 35.8 | 37.9 | 0.7 |
| 2017 | 10.8 | 89.2 | 3.3 | 31.3 | 51.1 | 3.5 |
| 2018 | 6.1 | 93.9 | 1.9 | 25.0 | 59.2 | 7.8 |
| 2019 | 4.0 | 96.0 | 1.1 | 21.6 | 61.8 | 11.5 |
| 2020 | 3.0 | 97.0 | 0.8 | 16.3 | 62.0 | 17.9 |
| 2021 | 2.1 | 97.9 | 0.5 | 14.6 | 66.9 | 15.9 |
| 2022 | 1.5 | 98.5 | 0.4 | 10.5 | 56.0 | 31.6 |

All values are expressed as percentage.

**Abbreviations**: VKA, vitamin K antagonist; DOAC, direct oral anticoagulant.

*****Sum of prescriptions attributable to dabigatran, rivaroxaban, apixaban, and edoxaban.

Table S7. Baseline characteristics of patients with chronic obstructive pulmonary disease newly prescribed an oral anticoagulant in the year following atrial fibrillation diagnosis between 2016 and 2022

|  | **VKA**  **(n= 1079)** | **DOAC**  **(n = 14,277)** | **Dabigatran**  **(n= 264)** | **Rivaroxaban**  **(n= 3421)** | **Apixaban**  **(n= 8662)** | **Edoxaban**  **(n = 1930)** |
| --- | --- | --- | --- | --- | --- | --- |
| **Age (years), mean (SD)** | 76.6 (8.0) | 78.0 (8.0) | 77.2 (7.3) | 77.1 (7.9) | 78.3 (8.0) | 78.4 (8.0) |
| **Sex** |  |  |  |  |  |  |
| Male | 670 (62.9) | 8181 (57.3) | 171 (64.8) | 2079 (60.8) | 4867 (56.2) | 866 (44.9) |
| Female | 409 (37.9) | 6096 (42.7) | 93 (35.2) | 1342 (39.2) | 3795 (43.8) | 1064 (55.1) |
| **Ethnicity^a^** |  |  |  |  |  |  |
| White | 1026 (95.1) | 13,567 (95.0) | 253 (95.8) | 3237 (94.6) | 8256 (95.3) | 1821 (94.4) |
| Black | 9 (0.8) | 89 (0.6) | S* | 21 (0.6) | 49 (0.6) | 18 (0.9) |
| Asian | 15 (1.4) | 174 (1.2) | S* | 49 (1.4) | 106 (1.2) | 18 (0.9) |
| Other | 6 (0.6) | 117 (0.8) | S* | 31 (0.9) | 68 (0.8) | 17 (0.9) |
| Missing | 24 (2.2) | 330 (2.3) | 8 (3.0) | 83 (2.4) | 183 (2.1) | 56 (2.9) |
| **Body mass index** |  |  |  |  |  |  |
| < 30 kg/m^2^ | 645 (59.8) | 8784 (61.5) | 147 (55.7) | 2067 (60.4) | 5350 (61.8) | 1220 (63.2) |
| ≥ 30kg/m^2^ | 408 (37.8) | 5074 (35.5) | 103 (39.0) | 1255 (36.7) | 3068 (35.4) | 648 (33.6) |
| Missing | 26 (2.4) | 419 (2.9) | 14 (5.3) | 99 (2.9) | 244 (2.8) | 62 (3.2) |
| **Smoking** |  |  |  |  |  |  |
| Non-smoker | 167 (15.5) | 1994 (14.0) | 28 (10.6) | 498 (14.6) | 1200 (13.9) | 268 (13.9) |
| Ex smoker | 561 (52.0) | 7387 (51.7) | 159 (60.2) | 1755 (51.3) | 4488 (51.7) | 985 (51.1) |
| Smoker | 342 (31.7) | 4780 (33.5) | 75 (28.4) | 1143 (33.4) | 2903 (33.6) | 659 (34.1) |
| Missing | 9 (0.8) | 116 (0.8) | S* | 25 (0.7) | 71 (0.8) | 18 (0.9) |
| **Alcohol abuse** | 108 (10.0) | 1545 (10.8) | 17 (6.4) | 368 (10.8) | 965 (11.1) | 195 (10.1) |
| **Moderate COPD exacerbation^b^**** | 559 (51.8) | 6702 (46.9) | 131 (49.6) | 1682 (49.2) | 4095 (47.3) | 794 (41.1) |
| **Hospitalization for COPD**** |  |  |  |  |  |  |
| 0 | 956 (88.6) | 12,297 (86.1) | 240 (90.9) | 2968 (86.8) | 7359 (85.0) | 1730 (89.6) |
| 1 | 91 (8.4) | 1520 (10.7) | 22 (8.3) | 343 (10.0) | 1002 (11.6) | 153 (7.9) |
| ≥ 2 | 32 (3.0) | 460 (3.2) | 2 (0.8) | 110 (3.2) | 301 (3.5) | 47 (2.4) |
| **Hospitalization for pneumonia**** | 263 (24.4) | 3919 (27.5) | 52 (19.7) | 849 (24.8) | 2538 (29.3) | 480 (24.9) |
| **Severity of dyspnea***** |  |  |  |  |  |  |
| No/mild dyspnea | 369 (34.2) | 4920 (34.5) | 92 (34.9) | 1255 (36.7) | 2909 (33.6) | 664 (34.4) |
| Moderate/severe dyspnea | 623 (57.5) | 8141 (57.0) | 157 (59.5) | 1884 (55.1) | 4993 (57.6) | 1107 (57.4) |
| Missing | 87 (8.1) | 1216 (8.5) | 15 (5.7) | 282 (8.3) | 760 (8.8) | 159 (8.2) |
| **FEV_1_ (% predicted), mean (SD)***** | 62.1 (18.9) | 62.4 (19.6) | 62.0 (18.5) | 62.2 (19.6) | 61.9 (19.6) | 65.2 (19.8) |
| < 50 | 240 (22.2) | 3047 (21.3) | 59 (22.4) | 777 (22.7) | 1909 (22.0) | 302 (15.6) |
| 50 -< 80 | 499 (46.2) | 5749 (40.3) | 117 (44.3) | 1504 (44.0) | 3466 (40.0) | 662 (34.3) |
| 80-120 | 160 (14.8) | 2051 (14.4) | 37 (14.0) | 522 (15.3) | 1192 (13.8) | 300 (15.6) |
| Missing | 180 (16.7) | 3430 (24.0) | 51 (19.3) | 618 (18.0) | 2095 (24.2) | 666 (34.5) |
| **Blood eosinophil count cells/µl, mean (SD)** | 237.5 (197.0) | 229.7 (220.7) | 248.8 (254.9) | 228.3 (202.9) | 229.1 (223.7) | 232.2 (231.9) |
| < 150 | 385 (35.7) | 5306 (37.2) | 86 (32.6) | 1250 (36.6) | 3255 (37.6) | 715 (37.0) |
| 150-300 | 414 (38.4) | 5457 (38.2) | 104 (39.4) | 1322 (38.6) | 3279 (37.9) | 752 (39.0) |
| > 300 | 203 (18.8) | 2519 (17.6) | 56 (21.2) | 602 (17.6) | 1532 (17.7) | 329 (17.1) |
| Missing | 77 (7.1) | 995 (7.0) | 18 (6.8) | 247 (7.2) | 596 (6.9) | 134 (6.9) |
| **Respiratory medications**** |  |  |  |  |  |  |
| LABA | 723 (67.0) | 10,002 (70.1) | 181 (68.6) | 2381 (69.6) | 6091 (70.3) | 1349 (69.9) |
| LAMA | 632 (58.6) | 9191 (64.4) | 171 (64.8) | 2189 (64.0) | 5569 (64.3) | 1262 (65.4) |
| Short-acting inhaled beta agonists | 877 (81.3) | 11,353 (79.5) | 205 (77.7) | 2735 (80.0) | 6899 (79.7) | 1514 (78.5) |
| Short-acting antimuscarinics | 54 (5.0) | 617 (4.3) | 13 (4.9) | 168 (4.9) | 372 (4.3) | 64 (3.3) |
| Inhaled corticosteroids | 708 (65.6) | 8992 (63.0) | 167 (63.3) | 2200 (64.3) | 5464 (63.1) | 1161 (60.2) |
| Methylxanthines | 51 (4.7) | 617 (4.3) | 14 (5.3) | 140 (4.1) | 391 (4.5) | 72 (3.7) |
| Antibiotics for respiratory conditions | 806 (74.7) | 9777 (68.5) | 181 (68.6) | 2391 (69.9) | 5983 (69.1) | 1222 (63.3) |
| **Comorbidities** |  |  |  |  |  |  |
| Hypertension | 939 (87.0) | 12,242 (85.8) | 220 (83.3) | 2906 (85.0) | 7475 (86.3) | 1641 (85.0) |
| Diabetes | 400 (37.1) | 5091 (35.7) | 89 (33.7) | 1201 (35.1) | 3061 (35.3) | 740 (38.5) |
| Coronary artery disease^c^ | 472 (43.7) | 5677 (39.8) | 95 (36.0) | 1328 (38.8) | 3511 (40.5) | 743 (38.5) |
| Myocardial infarction | 247 (22.9) | 3086 (21.6) | 46 (17.4) | 707 (20.7) | 1926 (22.2) | 407 (21.1) |
| Congestive heart failure | 407 (37.7) | 4683 (32.8) | 83 (31.4) | 988 (28.9) | 3021 (34.9) | 591 (30.6) |
| Ischaemic stroke/TIA | 164 (15.2) | 2617 (18.3) | 74 (28.0) | 514 (15.0) | 1684 (19.4) | 345 (17.9) |
| Systemic embolism | 12 (1.1) | 122 (0.9) | S* | 25 (0.7) | 82 (1.0) | 14 (0.7) |
| Peripheral arterial disease | 294 (27.3) | 3619 (25.4) | 69 (26.1) | 803 (23.5) | 2292 (26.5) | 455 (23.6) |
| Chronic kidney disease | 420 (38.9) | 4553 (31.9) | 73 (27.7) | 970 (28.4) | 2881 (33.3) | 629 (32.5) |
| Liver disease | 80 (7.4) | 1074 (7.5) | 11 (4.2) | 254 (7.4) | 679 (7.8) | 130 (6.7) |
| Cancer^d^ | 255 (23.6) | 3712 (26.0) | 78 (29.6) | 842 (26.6) | 2259 (26.1) | 533 (27.6) |
| History of bleeding | 522 (48.4) | 7122 (49.9) | 110 (41.7) | 1635 (47.8) | 4371 (50.5) | 1006 (52.1) |
| Dementia | 52 (4.8) | 1229 (8.6) | 14 (5.3) | 214 (6.3) | 811 (9.4) | 190 (9.8) |
| Obstructive sleep apnea | 64 (5.9) | 844 (5.9) | 17 (6.4) | 208 (6.1) | 510 (5.9) | 109 (5.7) |
| **Medications**** |  |  |  |  |  |  |
| Antiplatelet agents | 557 (51.6) | 6607 (46.3) | 134 (50.8) | 1598 (46.7) | 4046 (46.7) | 829 (43.0) |
| Calcium channel blockers | 476 (44.1) | 5958 (41.7) | 103 (39.0) | 1446 (42.3) | 3602 (41.6) | 807 (41.8) |
| Angiotensin-converting enzyme inhibitors | 426 (39.5) | 5263 (36.9) | 88 (33.3) | 1289 (37.7) | 3203 (37.0) | 683 (35.4) |
| Angiotensin receptor blockers | 218 (20.2) | 2764 (19.4) | 60 (22.7) | 651 (19.0) | 1677 (19.4) | 376 (19.5) |
| Thiazides | 170 (15.8) | 2144 (15.0) | 48 (18.2) | 555 (16.2) | 1271 (14.7) | 270 (14.0) |
| Beta blockers | 628 (58.2) | 8655 (60.6) | 151 (57.2) | 1987 (58.1) | 5336 (61.6) | 1181 (61.2) |
| Nonsteroidal anti-inflammatory drugs | 108 (10.0) | 1223 (8.6) | 28 (10.6) | 317 (9.3) | 730 (8.4) | 148 (7.7) |
| Statins or other lipid lowering drugs | 690 (64.0) | 9108 (63.8) | 174 (65.9) | 2115 (61.8) | 5616 (64.8) | 1203 (62.3) |
| **Number of Hospitalizations**** |  |  |  |  |  |  |
| 0 | 334 (30.9) | 3596 (25.2) | 95 (36.0) | 1017 (29.7) | 1876 (21.7) | 608 (31.5) |
| 1 | 318 (29.5) | 5060 (35.4) | 90 (34.1) | 1136 (33.2) | 3200 (36.9) | 634 (32.9) |
| ≥ 2 | 427 (39.6) | 5621 (39.4) | 79 (29.9) | 1268 (37.1) | 3586 (41.4) | 688 (35.6) |
| **CHA_2_DS_2_-VA** |  |  |  |  |  |  |
| 0 | 9 (0.8) | 57 (0.4) | S* | 11 (0.3) | 34 (0.4) | 11 (0.6) |
| 1 | 46 (4.3) | 576 (4.0) | S* | 170 (5.0) | 323 (3.7) | 73 (3.8) |
| ≥ 2 | 1024 (94.9) | 13,644 (95.6) | 253 (95.8) | 3240 (94.7) | 8305 (95.9) | 1846 (95.7) |
| **Modified HAS-BLED** |  |  |  |  |  |  |
| ≤ 2 | 220 (20.4) | 3298 (23.1) | 71 (26.9) | 855 (25.0) | 1904 (22.0) | 468 (24.3) |
| > 2 | 859 (79.6) | 10,979 (76.9) | 193 (73.1) | 2566 (75.0) | 6758 (78.0) | 1462 (75.7) |

All values are expressed as n (%) and measured at any time prior to initial OAC prescription within the year following AF diagnosis, unless otherwise specified.

*Cells with less than five events were suppressed due to privacy restrictions.

**^a^**Ethnicity **–** Black: African, Caribbean, any other Black, African, or Caribbean background; Asian: Indian, Pakistani, Bangladeshi, Chinese, any other Asian; Other: Mixed, any other ethnic group.

**^b^**Moderate COPD exacerbation was defined as a prescription for prednisolone in the prior year.

**^c^**Coronary artery disease includes myocardial infarction and ischaemic heart disease.

**^d^**Cancer includes all cancers except non-melanoma skin cancer.

******Measured in the year before the patients initial OAC prescription within the year following AF diagnosis.

*******Most recent measure in the prior 3 years.

**Acronyms/abbreviations –** CHA_2_DS_2_-VA, congestive heart failure, hypertension, age ≥75 years, diabetes mellitus, stroke/TIA, vascular disease, age 65–74 years; modified HAS-BLED, hypertension, abnormal renal and/or liver function, stroke/TIA, history of bleeding, age >65 years, antiplatelet/nonsteroidal anti-inflammatory drug use or alcohol abuse; VKA, vitamin K antagonist; DOAC, direct oral anticoagulant; SD, standard deviation; FEV_1_, forced expiratory volume in one second; LAMA, long-acting muscarinic antagonist; LABA, long-acting beta agonist; TIA, transient ischaemic attack.

Table S8. Hazard ratios and 95% confidence intervals for the association between patient characteristics and the initiation of an oral anticoagulant in the year following atrial fibrillation diagnosis in patients with chronic obstructive pulmonary disease in UK primary care from 2010 to 2022, conditioned on calendar year

|  | **OAC initiated^a^,**  **n =23,096** | **No OAC initiated^a^,**  **n = 11,757** | **Crude HR** | **Adjusted* HR (95% CI)** |
| --- | --- | --- | --- | --- |
| **Age (years)** |  |  |  |  |
| 50-59 | 374 (1.6) | 267 (2.3) | 1.00 (ref.) | 1.00 (ref.) |
| 60-69 | 3377 (14.6) | 1625 (13.8) | 1.40 | 1.40 (1.26, 1.56) |
| 70-79 | 9682 (41.9) | 4054 (34.5) | 1.54 | 1.57 (1.42, 1.74) |
| 80-89 | 8368 (36.2) | 4522 (38.5) | 1.37 | 1.46 (1.32, 1.62) |
| ≥90 | 1293 (5.6) | 1289 (11.0) | 0.96 | 1.09 (0.97, 1.22) |
| **Sex** |  |  |  |  |
| Female | 9640 (41.7) | 5089 (43.3) | 1.00 (ref.) | 1.00 (ref.) |
| Male | 13,454 (58.3) | 6668 (56.7) | 1.02 | 1.01 (0.98, 1.04) |
| **Ethnicity** |  |  |  |  |
| White | 22,079 (95.6) | 11,269 (95.8) | 1.00 (ref.) | 1.00 (ref.) |
| Other^b^ | 550 (2.4) | 288 (2.4) | 0.87 | 0.88 (0.81, 0.96) |
| Missing**†** | 467 (2.0) | 200 (1.7) | 1.13 | 1.03 (0.94, 1.14) |
| **Comorbidities** |  |  |  |  |
| Congestive heart failure | 6817 (29.5) | 3878 (33.0) | 0.90 | 0.96 (0.93, 0.99) |
| Hypertension | 19,409 (84.0) | 9648 (82.1) | 1.09 | 1.15 (1.11, 1.19) |
| Myocardial infarction | 4733 (20.5) | 2608 (22.2) | 0.92 | 0.95 (0.92, 0.98) |
| Peripheral arterial disease | 5634 (24.4) | 3200 (27.2) | 0.90 | 0.92 (0.89, 0.94) |
| Stroke or TIA | 4019 (17.4) | 2016 (17.2) | 1.02 | 1.06 (1.02, 1.09) |
| Systemic embolism | 173 (0.8) | 115 (1.0) | 0.76 | 0.84 (0.73, 0.97) |
| Diabetes | 7519 (32.6) | 3430 (29.2) | 1.02 | 1.05 (1.02, 1.08) |
| Chronic kidney disease | 7224 (31.3) | 4132 (35.2) | 0.91 | 0.94 (0.92, 0.97) |
| Liver disease | 1398 (6.1) | 821 (7.0) | 0.82 | 0.82 (0.78, 0.87) |
| History of bleeding | 10,622 (46.0) | 5872 (49.9) | 0.86 | 0.89 (0.86, 0.91) |
| Cancer^c^ | 5396 (23.4) | 3403 (28.9) | 0.84 | 0.85 (0.83, 0.88) |
| Dementia | 1463 (6.3) | 1536 (13.1) | 0.61 | 0.66 (0.63, 0.70) |
| Obstructive sleep apnoea | 1213 (5.3) | 466 (4.0) | 1.07 | 1.09 (1.02, 1.15) |
| **Level of dyspnea**** |  |  |  |  |
| No-mild | 7982 (34.6) | 3448 (29.3) | 1.00 (ref.) | 1.00 (ref.) |
| Moderate-severe | 13,094 (56.7) | 7195 (61.2) | 0.90 | 0.96 (0.93, 0.99) |
| Missing**†** | 2020 (8.7) | 1114 (9.5) | 0.90 | 1.03 (0.98, 1.09) |
| **Percent predicted FEV_1_**** |  |  |  |  |
| FEV_1_ ≥80% | 3220 (13.9) | 1246 (10.6) | 1.00 (ref.) | 1.00 (ref.) |
| FEV_1_ 50 - <80% | 9920 (42.9) | 4386 (37.3) | 0.96 | 0.97 (0.94, 1.01) |
| FEV_1_ <50% | 5400 (23.4) | 3338 (28.4) | 0.85 | 0.88 (0.84, 0.92) |
| Missing**†** | 4556 (19.7) | 2787 (23.7) | 0.79 | 0.84 (0.80, 0.88) |
| **Prednisolone prescription***** | 10,917 (47.3) | 5386 (45.8) | 1.05 | 1.08 (1.05, 1.11) |
| **Number of COPD hospitalizations***** | |  |  |  |
| 0 | 20,545 (89.0) | 9922 (84.4) | 1.00 (ref.) | 1.00 (ref.) |
| 1 | 1964 (8.5) | 1299 (11.5) | 0.86 | 0.89 (0.85, 0.93) |
| ≥2 | 585 (2.5) | 536 (4.6) | 0.72 | 0.75 (0.69, 0.81) |

Age was measured at the time of AF diagnosis, while all other covariates were measured at any time prior, unless otherwise specified.

**^a^**Values expressed as n (%).

**^b^**Other includes individuals identifying as Black African, Black Caribbean, any other Black, African, or Caribbean background, Indian, Pakistani, Bangladeshi, Chinese, any other Asian background, Mixed, or any other ethnic group.

**^c^**Cancer includes all cancers except non-melanoma skin cancer.

†Patients with missing ethnicity, dyspnea, and percent predicted FEV_1_ recordings, were retained by including a separate category for missing values in the Cox proportional hazards regression models.

*****Adjusted for all variables listed in the table.

******Most recent measure in the prior 3 years.

*******Measured in the year before AF diagnosis.

**Abbreviations**: HR, hazard ratio; CI, confidence interval; OAC, oral anticoagulant; COPD, chronic obstructive pulmonary disease; TIA, transient ischaemic attack; FEV_1_, percent predicted forced expiratory volume in one second; ref., reference.

Table S9. Odds ratios and 95% confidence intervals for the association between patient characteristics and the initiation of direct oral anticoagulants compared to the initiation of vitamin K antagonists in the year following atrial fibrillation diagnosis in patients with chronic obstructive pulmonary disease in UK primary care from 2010 to 2022, conditioned on calendar year

|  | **DOAC initiated^a^,**  **n =16,233** | **VKA initiated^a^,**  **n = 6861** | **Crude OR** | **Adjusted^*^ OR (95% CI)** |
| --- | --- | --- | --- | --- |
| **Age (years)** |  |  |  |  |
| 50-59 | 231 (1.4) | 132 (1.9) | 1.00 (ref.) | 1.00 (ref.) |
| 60-69 | 2179 (13.4) | 1156 (16.9) | 1.52 | 1.51 (1.05, 2.16) |
| 70-79 | 6654 (41.0) | 2980 (43.4) | 1.65 | 1.63 (1.15, 2.31) |
| 80-89 | 6058 (37.3) | 2370 (34.5) | 1.80 | 1.80 (1.27, 2.56) |
| ≥90 | 1111 (6.8) | 223 (3.3) | 2.82 | 2.86 (1.92, 4.28) |
| **Sex** |  |  |  |  |
| Female | 6909 (42.6) | 2731 (39.8) | 1.00 (ref.) | 1.00 (ref.) |
| Male | 9324 (57.4) | 4130 (60.2) | 0.91 | 0.93 (0.85, 1.02) |
| **Ethnicity** |  |  |  |  |
| White | 15,451 (95.2) | 6628 (96.6) | 1.00 (ref.) | 1.00 (ref.) |
| Other^b^ | 411 (2.5) | 139 (2.0) | 0.84 | 0.90 (0.67, 1.21) |
| Missing† | 371 (2.3) | 94 (1.4) | 1.26 | 1.25 (0.89, 1.76) |
| **Comorbidities** |  |  |  |  |
| Congestive heart failure | 5305 (32.7) | 2323 (33.9) | 0.87 | 0.86 (0.78, 0.96) |
| Hypertension | 13,953 (86.0) | 6013 (87.6) | 0.96 | 1.04 (0.90, 1.19) |
| Myocardial infarction | 3502 (21.6) | 1367 (19.9) | 0.97 | 1.04 (0.93, 1.17) |
| Peripheral arterial disease | 4127 (25.4) | 1644 (24.0) | 1.00 | 1.03 (0.93, 1.15) |
| Stroke or TIA | 3035 (18.7) | 1167 (17.0) | 1.39 | 1.40 (1.25, 1.58) |
| Systemic embolism | 136 (0.8) | 51 (0.7) | 0.84 | 0.82 (0.50, 1.35) |
| Diabetes | 5640 (34.7) | 2047 (29.8) | 0.86 | 0.91 (0.82, 1.00) |
| Chronic kidney disease | 5115 (31.5) | 2231 (32.5) | 0.79 | 0.74 (0.67, 0.82) |
| Liver disease | 1141 (7.0) | 286 (4.2) | 0.80 | 0.86 (0.70, 1.04) |
| History of bleeding | 7957 (49.0) | 2854 (41.6) | 0.98 | 0.98 (0.90, 1.08) |
| Cancer^c^ | 4130 (25.4) | 1355 (19.8) | 1.04 | 1.02 (0.91, 1.13) |
| Dementia | 1344 (8.3) | 256 (3.7) | 1.54 | 1.40 (1.14, 1.73) |
| Obstructive sleep apnea | 923 (5.7) | 321 (4.7) | 0.80 | 0.91 (0.74, 1.12) |
| **Level of dyspnea**** |  |  |  |  |
| No-mild | 5555 (34.2) | 2290 (33.4) | 1.00 (ref.) | 1.00 (ref.) |
| Moderate-severe | 9300 (57.3) | 4037 (58.8) | 1.02 | 0.99 (0.89, 1.09) |
| Missing† | 1378 (8.5) | 534 (7.8) | 1.07 | 1.00 (0.81, 1.23) |
| **Percent predicted FEV_1_**** | | | | |
| FEV_1_ ≥80% | 3609 (22.2) | 1847 (26.9) | 1.00 (ref.) | 1.00 (ref.) |
| FEV_1_ 50 - <80% | 6628 (40.8) | 3233 (47.1) | 1.04 | 1.03 (0.90, 1.18) |
| FEV_1_ <50% | 2279 (14.0) | 893 (13.0) | 1.23 | 1.18 (1.01, 1.37) |
| Missing† | 3717 (22.9) | 888 (12.9) | 1.21 | 1.14 (0.95, 1.38) |
| **Prednisolone prescription***** | 7721 (47.6) | 3339 (48.7) | 1.00 | 0.99 (0.90, 1.09) |
| **Number of COPD hospitalizations***** | | | | |
| 0 | 13,941 (86.0) | 5938 (86.6) | 1.00 (ref.) | 1.00 (ref.) |
| 1 | 1728 (10.6) | 706 (10.3) | 1.18 | 1.19 (1.03, 1.38) |
| ≥2 | 564 (3.5) | 217 (3.2) | 1.51 | 1.51 (1.18, 1.95) |

Age was measured at the time of the patients initial OAC prescription following AF diagnosis, while all other covariates were measured at any time prior, unless otherwise specified.

**^a^**Values expressed as n (%).

**^b^**Other includes individuals identifying as Black African, Black Caribbean, any other Black, African, or Caribbean background, Indian, Pakistani, Bangladeshi, Chinese, any other Asian background, Mixed, or any other ethnic group.

**^c^**Cancer includes all cancers except non-melanoma skin cancer.

**†**Patients with missing ethnicity, dyspnea, and percent predicted FEV_1_ recordings, were retained by including a separate category for missing values in the logistic regression models.

*****Adjusted for all variables listed in the table.

******Most recent measure in the prior 3 years.

*******Measured in the year before the patients initial OAC prescription within the year following AF diagnosis.

**Abbreviations**: OR, odds ratio; CI, confidence interval; DOAC, direct oral anticoagulant; VKA, vitamin K antagonist; COPD, chronic obstructive pulmonary disease; TIA, transient ischaemic attack; FEV_1_, percent predicted forced expiratory volume in one second; ref., reference.

Figure S1. Flowchart of patient selection in study cohort of patients with chronic obstructive pulmonary disease in UK primary care (2010-2022)

**554,316**	Patients contributing data to CPRD between 1 January 2010 and 31 December 2022 with a diagnosis of COPD (before or during the study period), eligible to HES linkage

**59,830**	Excluded

**15,081**	< 1 year of medical information in the CPRD prior to cohort entry

**44,749**	COPD diagnosis before age 50 years

**494,486**	Eligible patients with COPD

**49,818**	Excluded patients with a diagnosis of AF prior to cohort entry

**444,668**	Study cohort of patients with COPD and no prior AF

AF

CPRD, Clinical Practice Research Datalink; COPD, chronic obstructive pulmonary disease; HES, Hospital Episodes Statistics; AF, atrial fibrillation.

Figure S2. Crude and standardized (to the age and sex distribution of the study cohort in the year 2022) incidence rates of atrial fibrillation per 1,000 person-years, in patients with chronic obstructive pulmonary disease in UK primary care from 2010 to 2022

Incidence Rate per 1000 Person-years

| Year | Crude | Standardized |
| --- | --- | --- |
| 2010 | 13.8192 | 14.0929 |
| 2011 | 14.5017 | 14.92 |
| 2012 | 15.3969 | 15.8536 |
| 2013 | 15.7231 | 16.2632 |
| 2014 | 15.9939 | 16.4813 |
| 2015 | 17.1218 | 17.7103 |
| 2016 | 17.5665 | 18.0914 |
| 2017 | 18.0115 | 18.4518 |
| 2018 | 17.8129 | 18.1902 |
| 2019 | 17.9678 | 18.2553 |
| 2020 | 14.0807 | 14.294 |
| 2021 | 16.8668 | 16.9547 |
| 2022 | 18.342 | 18.342 |

Figure S3. Crude incidence rates of atrial fibrillation per 1,000 person-years overall and stratified by sex, in patients with chronic obstructive pulmonary disease in UK primary care from 2010 to 2022

Incidence Rate per 1000 Person-years

| Year | Overall | Male | Female |
| --- | --- | --- | --- |
| 2010 | 13.8192 | 15.4939 | 12.0266 |
| 2011 | 14.5017 | 16.4184 | 12.4685 |
| 2012 | 15.3969 | 17.4159 | 13.2666 |
| 2013 | 15.7231 | 17.403 | 13.9547 |
| 2014 | 15.9939 | 18.2021 | 13.6782 |
| 2015 | 17.1218 | 19.9427 | 14.1875 |
| 2016 | 17.5665 | 20.0672 | 14.9747 |
| 2017 | 18.0115 | 20.4878 | 15.4572 |
| 2018 | 17.8129 | 20.1669 | 15.393 |
| 2019 | 17.9678 | 20.8314 | 15.0387 |
| 2020 | 14.0807 | 16.1685 | 11.9719 |
| 2021 | 16.8668 | 18.9678 | 14.7641 |
| 2022 | 18.342 | 21.2571 | 15.4497 |

Figure S4. Crude incidence rates of atrial fibrillation per 1,000 person-years, stratified by age, in patients with chronic obstructive pulmonary disease in UK primary care from 2010 to 2022

Incidence Rate per 1000 Person-years

| Year | Age ≥90 years | Age 80-89 years | Age 70-79 years | Age 60-69 years | Age 50-59 years |
| --- | --- | --- | --- | --- | --- |
| 2010 | 28.9892 | 24.623 | 14.7816 | 7.27052 | 3.42072 |
| 2011 | 34.1716 | 25.137 | 16.2215 | 7.29094 | 2.97227 |
| 2012 | 37.8862 | 27.7634 | 17.0296 | 7.3634 | 2.46152 |
| 2013 | 28.7159 | 28.7311 | 18.0499 | 7.66967 | 2.57697 |
| 2014 | 34.743 | 29.9194 | 17.2957 | 7.7105 | 3.37539 |
| 2015 | 39.9768 | 32.974 | 18.219 | 8.06811 | 3.09367 |
| 2016 | 42.6154 | 32.4901 | 18.7111 | 8.47492 | 3.79807 |
| 2017 | 43.5643 | 33.9061 | 19.0319 | 8.26545 | 3.44656 |
| 2018 | 44.6941 | 33.1936 | 18.4959 | 8.34457 | 3.6999 |
| 2019 | 37.4322 | 34.1037 | 18.3934 | 8.90889 | 3.72363 |
| 2020 | 34.8565 | 26.7476 | 14.0742 | 6.61422 | 3.18795 |
| 2021 | 44.3546 | 31.3567 | 17.0153 | 7.24266 | 3.85266 |
| 2022 | 42.616 | 34.8026 | 18.266 | 7.95814 | 4.30621 |

Figure S5. Crude incidence rates of atrial fibrillation per 1,000 person-years, stratified by ethnicity, in patients with chronic obstructive pulmonary disease in UK primary care from 2010 to 2022

Incidence Rate per 1000 Person-years

| Year | White | Other* |
| --- | --- | --- |
| 2010 | 14.3807 | 7.02911 |
| 2011 | 15.0964 | 8.45107 |
| 2012 | 16.0504 | 9.58977 |
| 2013 | 16.4232 | 7.84223 |
| 2014 | 16.8501 | 7.63627 |
| 2015 | 17.9991 | 11.1767 |
| 2016 | 18.3842 | 12.1897 |
| 2017 | 18.9445 | 12.7129 |
| 2018 | 18.9317 | 12.0077 |
| 2019 | 19.1229 | 13.0662 |
| 2020 | 15.2035 | 8.6582 |
| 2021 | 18.0139 | 12.1625 |
| 2022 | 19.6539 | 11.0068 |

^*^Other includes individuals identifying as Black African, Black Caribbean, any other Black, African, or Caribbean background, Indian, Pakistani, Bangladeshi, Chinese, any other Asian background, Mixed, or any other ethnic group.

Follow-up intervals for patients with missing ethnicity, including 345 person-years (5.5% of total person-time) and 710 corresponding events (1.9% of total events), were excluded.

Figure S6. Crude incidence rates of atrial fibrillation per 1,000 person-years, stratified by geographic region of practice, in patients with chronic obstructive pulmonary disease in UK primary care from 2010 to 2022

Incidence rate per 1000 person-years

| Year | North East | North West | Yorkshire and The Humber | East Midlands | West Midlands | East of England | London | South East | South West |
| --- | --- | --- | --- | --- | --- | --- | --- | --- | --- |
| 2010 | 13.1428 | 13.585 | 14.0457 | 12.2637 | 13.3542 | 16.659 | 13.7193 | 13.9024 | 14.4811 |
| 2011 | 14.0789 | 13.2833 | 13.8235 | 12.6159 | 14.2422 | 15.7594 | 13.5246 | 16.221 | 16.072 |
| 2012 | 15.398 | 14.3343 | 17.2517 | 14.9654 | 15.4362 | 18.3269 | 15.2626 | 15.3376 | 16.2492 |
| 2013 | 17.2736 | 15.0554 | 16.4351 | 17.8275 | 16.4302 | 13.3073 | 14.3281 | 15.6406 | 17.0226 |
| 2014 | 13.3944 | 15.5508 | 18.1016 | 16.7957 | 15.6918 | 15.8581 | 14.8673 | 17.0134 | 17.1419 |
| 2015 | 14.8922 | 16.8707 | 15.1744 | 17.022 | 17.2024 | 18.2283 | 15.947 | 17.0439 | 19.9062 |
| 2016 | 16.8296 | 17.2657 | 16.6789 | 18.566 | 17.0084 | 17.0257 | 16.9657 | 18.7308 | 18.2806 |
| 2017 | 21.8765 | 17.1206 | 16.6818 | 17.2625 | 19.2508 | 15.6852 | 17.1042 | 18.1374 | 18.5315 |
| 2018 | 19.1754 | 16.6702 | 17.9141 | 16.4132 | 20.1146 | 15.1333 | 17.8378 | 18.2468 | 16.8329 |
| 2019 | 19.8096 | 17.4425 | 16.6252 | 16.9903 | 18.6135 | 19.1016 | 18.0777 | 18.8308 | 16.3151 |
| 2020 | 15.5093 | 13.6698 | 13.9495 | 12.0742 | 14.3132 | 14.8031 | 14.8023 | 13.9952 | 13.582 |
| 2021 | 16.335 | 16.9357 | 18.8479 | 15.9581 | 16.4421 | 14.6919 | 16.5368 | 17.3186 | 17.278 |
| 2022 | 17.4701 | 18.4924 | 19.3521 | 18.1297 | 18.4223 | 15.8244 | 17.4274 | 19.4071 | 18.0701 |

Figure S7. Crude incidence rates of atrial fibrillation per 1,000 person-years, stratified by level of dyspnea, in patients with chronic obstructive pulmonary disease in UK primary care from 2010 to 2022

Incidence Rate per 1000 Person-years

| Year | Moderate-severe dyspnea | No-mild dyspnea |
| --- | --- | --- |
| 2010 | 18.8673 | 9.85392 |
| 2011 | 20.484 | 9.24538 |
| 2012 | 20.3717 | 11.0975 |
| 2013 | 21.7007 | 10.5999 |
| 2014 | 22.9032 | 10.5801 |
| 2015 | 23.7574 | 11.4182 |
| 2016 | 24.9973 | 11.7547 |
| 2017 | 25.0835 | 12.3994 |
| 2018 | 24.4961 | 12.5919 |
| 2019 | 25.4141 | 12.0848 |
| 2020 | 18.9863 | 10.313 |
| 2021 | 23.6948 | 11.1924 |
| 2022 | 24.6369 | 13.3494 |

Follow-up intervals for patients with missing dyspnea recordings, including 653 person-years (10.4% of total person-time) and 3378 corresponding events (9.0% of total events), were excluded.

Figure S8. Crude incidence rates of atrial fibrillation per 1,000 person-years, stratified by the presence of severe chronic obstructive pulmonary disease (COPD) exacerbation in the prior year, in patients with COPD in UK primary care from 2010 to 2022

Incidence Rate per 1000 Person-years

| Year | Severe exacerbation | No severe exacerbation |
| --- | --- | --- |
| 2010 | 42.8982 | 12.1745 |
| 2011 | 43.6335 | 12.8006 |
| 2012 | 46.928 | 13.6744 |
| 2013 | 46.9174 | 13.9784 |
| 2014 | 49.7489 | 14.2247 |
| 2015 | 49.2828 | 15.3574 |
| 2016 | 52.5703 | 15.7394 |
| 2017 | 57.167 | 15.9429 |
| 2018 | 51.819 | 15.989 |
| 2019 | 55.0261 | 15.9901 |
| 2020 | 45.7069 | 12.6129 |
| 2021 | 66.5217 | 15.2497 |
| 2022 | 55.8331 | 16.7574 |

Figure S9. Crude incidence rates of atrial fibrillation per 1,000 person-years, stratified by the presence of moderate or severe chronic obstructive pulmonary disease (COPD) exacerbation in the prior year, in patients with COPD in UK primary care from 2010 to 2022

Incidence Rate per 1000 Person-years

| Year | Moderate or severe exacerbation | No moderate or severe exacerbation |
| --- | --- | --- |
| 2010 | 19.9038 | 10.6463 |
| 2011 | 20.0563 | 11.328 |
| 2012 | 20.2043 | 12.4755 |
| 2013 | 20.9273 | 12.3162 |
| 2014 | 21.8563 | 12.1424 |
| 2015 | 22.2858 | 13.4567 |
| 2016 | 23.7055 | 13.3437 |
| 2017 | 23.7103 | 14.1069 |
| 2018 | 22.8313 | 14.4124 |
| 2019 | 24.051 | 13.9924 |
| 2020 | 18.1985 | 11.4253 |
| 2021 | 23.8708 | 13.77 |
| 2022 | 24.2658 | 15.2007 |

Figure S10. Crude incidence rates of atrial fibrillation per 1,000 person-years (2010-2022), stratified by level of dyspnea, age, and sex, in patients with chronic obstructive pulmonary disease in UK primary care

Incidence Rate per 1000 Person-years

| Age | Female no-mild dyspnea | Female moderate-severe dyspnea | Male no-mild dyspnea | Male moderate-severe dyspnea |
| --- | --- | --- | --- | --- |
| 50-59 years | 1.58 | 2.905 | 3.045 | 6.201 |
| 60-69 years | 3.809 | 8.103 | 6.699 | 14.08 |
| 70-79 years | 9.738 | 19.03 | 14.7 | 28.02 |
| 80-89 years | 20.85 | 33.62 | 25.16 | 42.25 |
| ≥90 years | 27.92 | 41.17 | 35.18 | 52.28 |

Follow-up intervals for patients with missing dyspnea recordings, including 653 person-years (10.4% of total person-time) and 3378 corresponding events (9.0% of total events), were excluded.

Figure S11. Crude incidence rates of atrial fibrillation per 1,000 person-years (2010-2022), stratified by percent predicted forced expiratory volume in one second (FEV_1_), age, and sex, in patients with chronic obstructive pulmonary disease in UK primary care

Incidence Rate per 1000 Person-years

| Age | Female FEV1 ≥ 80% | Female FEV1 50-79% | Female FEV1 < 50% | Male FEV1 ≥ 80% | Male FEV1 50-79% | Male FEV1 < 50% |
| --- | --- | --- | --- | --- | --- | --- |
| 50-59 years | 1.62429 | 1.87817 | 3.6534 | 2.91157 | 3.98982 | 5.51457 |
| 60-69 years | 3.7764 | 5.52316 | 6.93821 | 7.57083 | 9.42162 | 11.1469 |
| 70-79 years | 11.0247 | 13.7894 | 15.602 | 17.351 | 20.0745 | 22.0363 |
| 80-89 years | 23.8884 | 28.8383 | 29.0791 | 28.5826 | 33.8643 | 35.3002 |
| ≥ 90 years | 34.3883 | 38.2867 | 37.2751 | 43.9274 | 44.4154 | 46.1013 |

Follow-up intervals for patients with missing FEV_1_ recordings, including 1336 person-years (21.4% of total person-time) and 8783 corresponding events (23.3% of total events), were excluded.

Figure S12. Crude incidence rates of atrial fibrillation per 1,000 person-years (2010-2022), stratified by the presence of moderate or severe chronic obstructive pulmonary disease (COPD) exacerbation in the prior year, age, and sex, in patients with COPD in UK primary care

Incidence Rate per 1000 Person-years

| Age | Female no moderate or severe exacerbation | Female moderate or severe exacerbation | Male no moderate or severe exacerbation | Male moderate or severe exacerbation |
| --- | --- | --- | --- | --- |
| 50-59 years | 1.66438 | 2.95907 | 3.74761 | 6.35644 |
| 60-69 years | 4.1353 | 7.79905 | 7.48846 | 14.1652 |
| 70-79 years | 10.7984 | 18.7311 | 15.9178 | 28.2689 |
| 80-89 years | 21.77 | 36.7913 | 26.8575 | 45.4029 |
| ≥ 90 years | 30.0484 | 45.1701 | 36.5818 | 57.0076 |

Figure S13. Flowchart of patient selection in sub-cohort of patients with chronic obstructive pulmonary disease and newly diagnosed atrial fibrillation in UK primary care (2010-2022)

**37, 645**	Patients from study cohort with COPD and incident AF, diagnosed during follow-up

**2,794**	Excluded

**2770**	Patients with an OAC prescribed within the year before AF diagnosis

**24**	Patients with ≥ 2 first OAC prescriptions on the same day, within the year following AF diagnosis

**34, 851**	Sub-cohort of patients with COPD, newly diagnosed with AF

COPD, chronic obstructive pulmonary disease; AF, atrial fibrillation; OAC, oral anticoagulant.

Figure S14. Crude and standardized (to the age and sex distribution of the study cohort in the year 2022) rates of new users of oral anticoagulants in the year following atrial fibrillation diagnosis per 100 person-months, in patients with chronic obstructive pulmonary disease in UK primary care from 2010 to 2022

Incidence Rate per 100 Person-months

| Year | Crude | Standardized |
| --- | --- | --- |
| 2010 | 10.3281 | 11.8418 |
| 2011 | 7.07116 | 7.51496 |
| 2012 | 8.5288 | 9.184 |
| 2013 | 10.4462 | 9.184 |
| 2014 | 13.444 | 13.9848 |
| 2015 | 16.932 | 17.3575 |
| 2016 | 21.3867 | 20.9462 |
| 2017 | 22.8518 | 21.8708 |
| 2018 | 23.5735 | 23.3061 |
| 2019 | 27.4409 | 28.194 |
| 2020 | 24.5981 | 24.9657 |
| 2021 | 31.0788 | 31.5122 |
| 2022 | 30.1564 | 29.3919 |

Figure S15. Crude rates of new users of oral anticoagulants (OACs) in the year following atrial fibrillation diagnosis per 100 person-months overall, and stratified by direct oral anticoagulants (DOACs) and vitamin K antagonists (VKAs), in patients with chronic obstructive pulmonary disease in UK primary care from 2010 to 2022

Incidence Rate per 100 Person-months

| Year | OACs overall | DOACs | VKAs |
| --- | --- | --- | --- |
| 2010 | 10.3281 | 0 | 10.3281 |
| 2011 | 7.07116 | 0 | 7.07116 |
| 2012 | 8.5288 | 0.462803 | 8.066 |
| 2013 | 10.4462 | 1.78496 | 8.66119 |
| 2014 | 13.444 | 4.35773 | 9.08632 |
| 2015 | 16.932 | 9.94102 | 6.99101 |
| 2016 | 21.3867 | 16.7478 | 4.63892 |
| 2017 | 22.8518 | 20.3888 | 2.46301 |
| 2018 | 23.5735 | 22.1321 | 1.4414 |
| 2019 | 27.4409 | 26.3527 | 1.08821 |
| 2020 | 24.5981 | 23.8723 | 0.725847 |
| 2021 | 31.0788 | 30.4304 | 0.648374 |
| 2022 | 30.1564 | 29.6962 | 0.460197 |

Figure S16. Crude rates of new users of vitamin K antagonists (VKAs) and direct oral anticoagulants (DOACs) in the year following atrial fibrillation diagnosis per 100 person-months, stratified by sex, in patients with chronic obstructive pulmonary disease in UK primary care from 2010 to 2022

Incidence Rate per 100 Person-months

| Year | DOAC male | DOAC female | VKA male | VKA female |
| --- | --- | --- | --- | --- |
| 2010 | 0 | 0 | 11.4626 | 8.90772 |
| 2011 | 0 | 0 | 7.57854 | 6.41809 |
| 2012 | 0.457568 | 0.469518 | 8.71994 | 7.22722 |
| 2013 | 2.07834 | 1.40342 | 8.72635 | 8.57646 |
| 2014 | 4.66417 | 3.96159 | 9.47783 | 8.58022 |
| 2015 | 9.34981 | 10.8184 | 7.2461 | 6.61245 |
| 2016 | 16.8079 | 16.664 | 4.93146 | 4.23137 |
| 2017 | 20.5743 | 20.1376 | 2.71837 | 2.11721 |
| 2018 | 21.4892 | 23.0247 | 1.45958 | 1.41615 |
| 2019 | 26.0489 | 26.7753 | 1.25328 | 0.858527 |
| 2020 | 23.7944 | 23.9824 | 0.734254 | 0.713956 |
| 2021 | 27.8929 | 34.1916 | 0.651477 | 0.643775 |
| 2022 | 28.996 | 30.7197 | 0.478707 | 0.433142 |

Figure S17. Crude rates of new users of oral anticoagulants in the year following atrial fibrillation diagnosis per 100 person-months, stratified by sex and CHA_2_DS_2_-VA score, in patients with chronic obstructive pulmonary disease in UK primary care from 2010 to 2022

Incidence Rate per 100 Person-months

| Year | Female CHA2DS2-VA ≥ 2 | Male CHA2DS2-VA ≥ 2 | Female CHA2DS2-VA = 1 | Male CHA2DS2-VA = 1 | Female CHA2DS2-VA = 0 | Male CHA2DS2-VA = 0 |
| --- | --- | --- | --- | --- | --- | --- |
| 2010 | 9.049 | 11.4244 | 7.61373 | 11.9908 | 5.23833 | 11.5383 |
| 2011 | 6.50504 | 7.77663 | 5.405 | 5.69069 | 4.96521 | 5.4803 |
| 2012 | 7.78193 | 9.46236 | 7.86596 | 6.31751 | 1.23021 | 6.36162 |
| 2013 | 9.98805 | 11.3401 | 9.81161 | 6.67539 | 10.0685 | 3.49267 |
| 2014 | 12.3403 | 14.4549 | 18.1267 | 13.8956 | 13.0312 | 3.50929 |
| 2015 | 17.5672 | 16.7694 | 19.386 | 16.5324 | 6.57117 | 8.37386 |
| 2016 | 21.4931 | 22.6402 | 16.3878 | 15.4872 | 4.77596 | 6.23231 |
| 2017 | 22.9039 | 24.1612 | 19.9282 | 16.591 | 4.78813 | 8.66219 |
| 2018 | 24.8969 | 23.6641 | 21.378 | 17.5365 | 6.25252 | 7.87129 |
| 2019 | 27.8062 | 28.111 | 31.4512 | 20.9417 | 5.48102 | 11.3815 |
| 2020 | 25.4201 | 26.0611 | 17.546 | 12.3973 | 5.5939 | 4.89906 |
| 2021 | 35.9497 | 29.6088 | 23.7391 | 22.1721 | 10.4967 | 5.17149 |
| 2022 | 32.5125 | 30.619 | 20.2861 | 27.0491 | 7.50423 | 6.27492 |

Figure S18. Crude rates of new users of vitamin K antagonists (VKAs) and direct oral anticoagulants (DOACs) in the year following atrial fibrillation diagnosis per 100 person-months, stratified by age, in patients with chronic obstructive pulmonary disease in UK primary care from 2010 to 2022

Incidence Rate per 100 Person-months

| Year | ≥90 years DOAC | ≥90 years VKA | 80-89 years DOAC | 80-89 years VKA | 70-79 years DOAC | 70-79 years VKA | 60-69 years DOAC | 60-69 years VKA | 50-59 years DOAC | 50-59 years VKA |
| --- | --- | --- | --- | --- | --- | --- | --- | --- | --- | --- |
| 2010 | 0 | 2.10708 | 0 | 8.39333 | 0 | 13.0821 | 0 | 12.6531 | 0 | 17.9943 |
| 2011 | 0 | 2.11625 | 0 | 6.16979 | 0 | 8.73418 | 0 | 8.27108 | 0 | 5.81168 |
| 2012 | 0.143966 | 2.87932 | 0.372357 | 7.34916 | 0.739252 | 9.75402 | 0.294344 | 9.27183 | 0 | 8.93545 |
| 2013 | 0.854389 | 2.05053 | 1.56375 | 8.09589 | 1.96645 | 10.3138 | 2.48156 | 9.71506 | 1.68852 | 10.694 |
| 2014 | 3.49809 | 4.46978 | 4.44247 | 8.97748 | 4.36728 | 10.0952 | 4.5943 | 9.82832 | 4.64543 | 6.33468 |
| 2015 | 6.88632 | 4.21402 | 9.69123 | 6.12893 | 11.4302 | 8.27448 | 9.51767 | 7.99015 | 5.03016 | 5.86852 |
| 2016 | 12.4342 | 1.43049 | 17.3585 | 4.83898 | 18.4312 | 5.28521 | 15.51 | 5.00321 | 7.57027 | 1.80245 |
| 2017 | 16.8905 | 0.871767 | 21.3637 | 2.60533 | 22.3894 | 2.95761 | 17.7793 | 2.0358 | 9.33846 | 1.86769 |
| 2018 | 16.0054 | 0.732273 | 22.4917 | 1.26043 | 25.1809 | 1.6713 | 19.1222 | 1.52978 | 13.1637 | 3.07152 |
| 2019 | 19.9132 | 0.253672 | 25.6962 | 0.777765 | 29.8256 | 1.24273 | 25.3681 | 1.79069 | 14.7718 | 2.23815 |
| 2020 | 15.2055 | 0.146207 | 24.9702 | 0.697697 | 26.7392 | 0.998558 | 21.7882 | 0.457736 | 13.1273 | 0.846925 |
| 2021 | 24.807 | 0.465131 | 30.0151 | 0.618072 | 34.7691 | 0.635051 | 27.3739 | 1.01762 | 14.6633 | 0 |
| 2022 | 19.3421 | 0.251197 | 32.562 | 0.342358 | 34.2745 | 0.456486 | 23.4514 | 0.556161 | 11.7407 | 1.95679 |

Figure S19. Percent of patients newly prescribed an oral anticoagulant in the year following atrial fibrillation diagnosis, stratified by age and sex, in patients with chronic obstructive pulmonary disease in UK primary care 2010-2022

Percent of patients prescribed an OAC

| Age | VKA | Apixaban | Rivaroxaban | Edoxaban | Dabigatran |
| --- | --- | --- | --- | --- | --- |
| 50-59 years Female | 17.2249 | 20.5742 | 10.0478 | 4.30622 | 1.43541 |
| 50-59 years Male | 22.2222 | 19.4444 | 10.4167 | 4.39815 | 1.62037 |
| 60-69 years Female | 23.3634 | 24.2664 | 13.7133 | 4.90971 | 1.46727 |
| 60-69 years Male | 22.9721 | 21.7337 | 15.0774 | 4.42724 | 1.88854 |
| 70-79 years Female | 20.5497 | 28.8323 | 12.7652 | 6.19812 | 1.53108 |
| 70-79 years Male | 22.4414 | 25.9771 | 14.3235 | 5.42393 | 2.14071 |
| 80-89 years Female | 17.7554 | 27.7052 | 11.9598 | 5.96315 | 1.35678 |
| 80-89 years Male | 18.9306 | 27.1387 | 12.7746 | 5.49133 | 1.60405 |
| ≥90 years Female | 7.88504 | 25.4237 | 9.65365 | 5.74797 | 1.03169 |
| ≥90 years Male | 9.46939 | 28.4898 | 8.81633 | 5.79592 | 1.22449 |

Figure S20. Crude rates of new users of oral anticoagulants in the year following atrial fibrillation diagnosis per 100 person-months, stratified by ethnicity, in patients with chronic obstructive pulmonary disease in UK primary care from 2010 to 2022

Incidence Rate per 100 Person-months

| Year | White | Other* |
| --- | --- | --- |
| 2010 | 10.5113 | 5.96304 |
| 2011 | 7.01425 | 10.6374 |
| 2012 | 8.57616 | 7.91248 |
| 2013 | 10.4574 | 8.63023 |
| 2014 | 13.4528 | 12.6513 |
| 2015 | 16.97 | 13.1652 |
| 2016 | 21.486 | 14.4218 |
| 2017 | 22.9823 | 19.173 |
| 2018 | 23.6792 | 21.711 |
| 2019 | 27.5317 | 20.9529 |
| 2020 | 24.8188 | 16.2736 |
| 2021 | 30.9456 | 25.9133 |
| 2022 | 30.0951 | 24.3113 |

*Other includes individuals identifying as Black African, Black Caribbean, any other Black, African, or Caribbean background, Indian, Pakistani, Bangladeshi, Chinese, any other Asian background, Mixed, or any other ethnic group.

Follow-up intervals for patients with missing ethnicity, including 68 person-months (1.5% of total person-time) and 465 corresponding events (2.0% of total events), were excluded.

Figure S21. Crude rates of new users of oral anticoagulants in the year following atrial fibrillation diagnosis per 100 person-months, stratified by geographic region of practice, in patients with chronic obstructive pulmonary disease in UK primary care from 2010 to 2022

Incidence rate per 100 person-months

| Year | North East | North West | Yorkshire and The Humber | East Midlands | West Midlands | East of England | London | South East | South West |
| --- | --- | --- | --- | --- | --- | --- | --- | --- | --- |
| 2010 | 8.68885 | 8.9188 | 13.6429 | 9.59668 | 11.0456 | 11.1446 | 9.84731 | 9.24162 | 14.4928 |
| 2011 | 5.80991 | 7.29664 | 8.14769 | 5.75387 | 7.08623 | 5.24481 | 6.08453 | 7.09289 | 8.72552 |
| 2012 | 7.40142 | 8.29284 | 11.3426 | 9.48502 | 9.83365 | 8.2056 | 7.35251 | 8.01231 | 8.95034 |
| 2013 | 9.23596 | 8.89099 | 11.2264 | 8.67841 | 10.9179 | 9.7917 | 10.2783 | 10.9447 | 13.3381 |
| 2014 | 13.0449 | 12.7571 | 14.7914 | 13.5214 | 14.0223 | 12.7226 | 9.87358 | 13.0836 | 18.9562 |
| 2015 | 19.0085 | 17.5239 | 20.0554 | 15.8979 | 17.1595 | 23.2474 | 12.3079 | 17.5905 | 17.0775 |
| 2016 | 21.1127 | 20.7905 | 21.3222 | 19.0649 | 21.3684 | 22.4629 | 17.664 | 24.7757 | 22.4194 |
| 2017 | 22.4485 | 24.2558 | 20.9537 | 20.5415 | 23.114 | 36.8366 | 17.9726 | 21.7933 | 25.6573 |
| 2018 | 16.9132 | 26.7237 | 18.7555 | 28.6938 | 24.2025 | 32.3198 | 19.3656 | 23.0165 | 26.9527 |
| 2019 | 31.4826 | 28.429 | 22.3556 | 25.7136 | 28.5281 | 38.7644 | 20.8553 | 26.0224 | 34.1348 |
| 2020 | 23.3408 | 23.4171 | 21.6018 | 24.2377 | 28.0085 | 31.1818 | 22.1632 | 23.7738 | 27.2998 |
| 2021 | 31.418 | 32.2054 | 25.9611 | 36.7911 | 32.1234 | 31.6129 | 26.5911 | 31.3934 | 33.1376 |
| 2022 | 26.583 | 29.8451 | 38.4542 | 37.1093 | 30.7862 | 38.6431 | 24.8822 | 32.9298 | 28.9574 |

Figure S22. Crude rates of new users of vitamin K antagonists (VKAs) and direct oral anticoagulants (DOACs) in the year following atrial fibrillation diagnosis per 100 person-months, stratified by level of dyspnea, in patients with chronic obstructive pulmonary disease in UK primary care from 2010 to 2022

Incidence Rate per 100 Person-months

| Year | VKA no-mild dyspnea | VKA moderate-severe dyspnea | DOAC no-mild dyspnea | DOAC moderate-severe dyspnea |
| --- | --- | --- | --- | --- |
| 2010 | 10.3562 | 10.4109 | 0 | 0 |
| 2011 | 7.06111 | 7.1096 | 0 | 0 |
| 2012 | 8.95942 | 7.68695 | 0.452737 | 0.454991 |
| 2013 | 9.69822 | 8.33838 | 1.96389 | 1.63062 |
| 2014 | 9.97907 | 8.87919 | 4.59092 | 4.20017 |
| 2015 | 7.82811 | 6.42462 | 10.8299 | 9.60875 |
| 2016 | 4.92646 | 4.46383 | 18.4111 | 15.5831 |
| 2017 | 2.46334 | 2.44277 | 20.5278 | 20.2303 |
| 2018 | 1.61692 | 1.31753 | 24.9196 | 20.3373 |
| 2019 | 1.54926 | 0.868279 | 28.3911 | 25.217 |
| 2020 | 0.895543 | 0.609222 | 26.8256 | 22.1351 |
| 2021 | 0.53577 | 0.75867 | 31.4318 | 29.5392 |
| 2022 | 0.305428 | 0.476241 | 32.7244 | 28.1436 |

Follow-up intervals for patients with missing dyspnea recordings, including 264 person-months (6.1% of total person-time) and 1,871 corresponding events (8.1% of total events), were excluded.

Figure S23. Crude rates of new users of vitamin K antagonists (VKAs) and direct oral anticoagulants (DOACs) in the year following atrial fibrillation diagnosis per 100 person-months, stratified by percent predicted forced expiratory volume in one second (FEV_1_), in patients with chronic obstructive pulmonary disease in UK primary care from 2010 to 2022

Incidence rate per 100 person-months

| Year | VKA FEV1 ≥ 80% | VKA FEV1 50-79% | VKA FEV1 < 50% | DOAC FEV1 ≥ 80% | DOAC FEV1 50-79% | DOAC FEV1 < 50% |
| --- | --- | --- | --- | --- | --- | --- |
| 2010 | 9.95422 | 11.3246 | 10.2485 | 0 | 0 | 0 |
| 2011 | 7.88755 | 7.97944 | 6.70464 | 0 | 0 | 0 |
| 2012 | 9.76872 | 8.94403 | 7.84226 | 0.404223 | 0.532601 | 0.398337 |
| 2013 | 10.1556 | 10.6586 | 7.10586 | 1.99129 | 1.77016 | 1.76996 |
| 2014 | 11.0376 | 10.1073 | 8.81725 | 3.91403 | 5.08442 | 3.76601 |
| 2015 | 9.29899 | 8.10176 | 5.62677 | 11.5209 | 10.2236 | 10.3667 |
| 2016 | 6.26665 | 5.41802 | 4.01134 | 21.7489 | 17.809 | 15.3434 |
| 2017 | 2.47695 | 2.61121 | 2.25961 | 21.0916 | 20.8645 | 19.5705 |
| 2018 | 1.6462 | 1.68632 | 1.07358 | 25.6234 | 22.7527 | 19.7317 |
| 2019 | 1.61109 | 1.34539 | 0.54019 | 28.406 | 28.2262 | 24.8072 |
| 2020 | 0.943613 | 0.753325 | 0.381636 | 28.214 | 25.6131 | 18.9728 |
| 2021 | 0.537757 | 0.734144 | 0.583953 | 34.4164 | 32.7774 | 27.7378 |
| 2022 | 0.163865 | 0.456905 | 0.728552 | 29.0041 | 32.5708 | 35.5533 |

Follow-up intervals for patients with missing FEV_1_ recordings, including 973 person-months (22.6% of total person-time) and 5,029 corresponding events (21.8% of total events), were excluded.

Figure S24. Crude rates of new users of vitamin K antagonists (VKAs) and direct oral anticoagulants (DOACs) in the year following atrial fibrillation diagnosis per 100 person-months, stratified by the presence of severe chronic obstructive pulmonary disease (COPD) exacerbation in the prior year, in patients with COPD in UK primary care from 2010 to 2022

Incidence Rate per 100 Person-months

| Year | VKA no severe exacerbation | VKA severe exacerbation | DOAC no severe exacerbation | DOAC severe exacerbation |
| --- | --- | --- | --- | --- |
| 2010 | 10.6816 | 8.51503 | 0 | 0 |
| 2011 | 7.60672 | 5.01351 | 0 | 0 |
| 2012 | 8.45497 | 6.21457 | 0.509982 | 0.243708 |
| 2013 | 9.09254 | 6.7341 | 1.83873 | 1.54759 |
| 2014 | 9.5295 | 7.04326 | 4.59622 | 3.25447 |
| 2015 | 7.43358 | 4.92677 | 9.91849 | 10.1049 |
| 2016 | 5.1495 | 2.2299 | 17.2454 | 14.2714 |
| 2017 | 2.53029 | 2.16919 | 20.8033 | 18.6094 |
| 2018 | 1.48694 | 1.22218 | 23.0168 | 17.8439 |
| 2019 | 1.22135 | 0.456494 | 27.3041 | 21.9769 |
| 2020 | 0.667284 | 1.03595 | 24.4353 | 20.5464 |
| 2021 | 0.672617 | 0.475384 | 30.4811 | 30.1869 |
| 2022 | 0.504221 | 0.193061 | 30.206 | 26.739 |

Figure S25. Crude rates of new users of vitamin K antagonists (VKAs) and direct oral anticoagulants (DOACs) in the year following atrial fibrillation diagnosis per 100 person-months, stratified by the presence of moderate or severe chronic obstructive pulmonary disease (COPD) exacerbation in the prior year, in patients with COPD in UK primary care from 2010 to 2022

Incidence Rate Per 100 Person-months

| Year | VKA no moderate or severe exacerbation | VKA moderate or severe exacerbation | DOAC no moderate or severe exacerbation | DOAC moderate or severe exacerbation |
| --- | --- | --- | --- | --- |
| 2010 | 10.2723 | 10.3257 | 0 | 0 |
| 2011 | 7.38977 | 6.7885 | 0 | 0 |
| 2012 | 8.18358 | 7.9272 | 0.502902 | 0.423915 |
| 2013 | 8.88858 | 8.46429 | 1.82491 | 1.75073 |
| 2014 | 9.15003 | 9.05076 | 4.35119 | 4.37374 |
| 2015 | 6.91535 | 7.07007 | 9.12676 | 10.6706 |
| 2016 | 4.27625 | 4.94794 | 15.5329 | 17.7787 |
| 2017 | 2.15423 | 2.74717 | 19.6896 | 21.0616 |
| 2018 | 1.4676 | 1.41742 | 21.3821 | 22.9253 |
| 2019 | 1.05711 | 1.11939 | 26.1928 | 26.5639 |
| 2020 | 0.731464 | 0.717838 | 23.1359 | 24.5128 |
| 2021 | 0.545758 | 0.791487 | 28.9252 | 32.5542 |
| 2022 | 0.568521 | 0.329659 | 29.1429 | 30.4185 |

Figure S26. Crude rates of new users of oral anticoagulants in the year following atrial fibrillation diagnosis per 100 person-months (2010-2022), stratified by level of dyspnea, age, and sex, in patients with chronic obstructive pulmonary disease in UK primary care

Incidence Rate per 100 Person-months

| Age | Female no-mild dyspnea | Female moderate-severe dyspnea | Male no-mild dyspnea | Male moderate-severe dyspnea |
| --- | --- | --- | --- | --- |
| 50-59 years | 8.19008 | 11.839 | 12.8042 | 12.9038 |
| 60-69 years | 18.9817 | 18.0516 | 16.5303 | 16.3287 |
| 70-79 years | 21.043 | 19.9642 | 20.7694 | 19.2452 |
| 80-89 years | 19.5273 | 15.9669 | 20.7626 | 16.0782 |
| ≥90 years | 11.6125 | 10.3145 | 13.9027 | 11.5008 |

Follow-up intervals for patients with missing dyspnea recordings, including 264 person-months (6.1% of total person-time) and 1,871 corresponding events (8.1% of total events), were excluded.

Figure S27. Crude rates of new users of oral anticoagulants in the year following atrial fibrillation diagnosis per 100 person-months (2010-2022), stratified by percent predicted forced expiratory volume in one second (FEV_1_), age, and sex, in patients with chronic obstructive pulmonary disease in UK primary care

Incidence Rate per 100 Person-months

| Age | Female FEV1 ≥ 80% | Female FEV1 50-79% | Female FEV1 < 50% | Male FEV1 ≥ 80% | Male FEV1 50-79% | Male FEV1 < 50% |
| --- | --- | --- | --- | --- | --- | --- |
| 50-59 years | 14.0772 | 11.3765 | 8.58311 | 9.89204 | 15.5148 | 12.319 |
| 60-69 years | 23.6101 | 19.059 | 14.3453 | 19.5765 | 16.9876 | 14.3105 |
| 70-79 years | 23.4482 | 20.5194 | 18.1497 | 21.9127 | 21.6725 | 16.7526 |
| 80-89 years | 19.7908 | 18.1953 | 15.4991 | 21.3719 | 18.8352 | 14.3725 |
| ≥ 90 years | 15.1562 | 9.75599 | 12.2771 | 15.8634 | 13.522 | 9.65399 |

Follow-up intervals for patients with missing FEV_1_ recordings, including 973 person-months (22.6% of total person-time) and 5,029 corresponding events (21.8% of total events), were excluded.

Figure S28. Crude rates of new users of oral anticoagulants in the year following atrial fibrillation diagnosis per 100 person-months (2010-2022), stratified by the presence of moderate or severe chronic obstructive pulmonary disease (COPD) exacerbation in the prior year, age, and sex in patients with COPD in UK primary care

Incidence Rate per 100 Person-months

| Age | Female no moderate or severe exacerbation | Female moderate or severe exacerbation | Male no moderate or severe exacerbation | Male moderate or severe exacerbation |
| --- | --- | --- | --- | --- |
| 50-59 years | 9.53066 | 11.7965 | 12.7369 | 13.2178 |
| 60-69 years | 16.9195 | 18.9084 | 16.2427 | 17.0887 |
| 70-79 years | 20.5636 | 20.2266 | 20.029 | 20.1325 |
| 80-89 years | 16.7766 | 17.2753 | 17.7212 | 17.3718 |
| ≥ 90 years | 11.0294 | 9.40026 | 12.6044 | 11.6475 |

Figure S29. Prevalence of beta blocker, digitalis glycosides, calcium channel blocker and antiarrhythmic drug therapy use, and ablation procedures for atrial fibrillation, in the year following atrial fibrillation diagnosis, stratified by sex, in patients with chronic obstructive pulmonary disease in UK primary care from 2010 to 2022

Percent of patients prescribed

| Year | Beta blockers female | Beta blockers male | Digitalis glycosides female | Digitalis glycosides male | Calcium channel blockers female | Calcium channel blockers male | Antiarrhythmics female | Antiarrhythmics male | Ablation female | Ablation male |
| --- | --- | --- | --- | --- | --- | --- | --- | --- | --- | --- |
| 2010 | 30.81 | 37.94 | 42.91 | 37.1 | 25.23 | 22.43 | 9.3 | 10.12 | 0.23 | 1.1 |
| 2011 | 39.89 | 41.58 | 44.04 | 32.67 | 24.15 | 17.52 | 9.57 | 9.14 | 0.74 | 0.76 |
| 2012 | 45 | 48.04 | 38.42 | 29.59 | 20.69 | 15.97 | 6.86 | 7.3 | 0.38 | 1.17 |
| 2013 | 50.79 | 53.28 | 33.22 | 26.97 | 19.6 | 15.39 | 6.15 | 7.83 | 0.97 | 1.2 |
| 2014 | 54.44 | 54.62 | 34.15 | 23.78 | 16.9 | 13.23 | 6.18 | 7.74 | 0.52 | 0.94 |
| 2015 | 56.71 | 58.71 | 27.99 | 22.76 | 14.24 | 11.24 | 5.61 | 5.79 | 0.33 | 1.06 |
| 2016 | 60.59 | 59.53 | 24.16 | 19.91 | 13.01 | 10.44 | 6.32 | 6.05 | 0.59 | 0.91 |
| 2017 | 61.39 | 58.33 | 26.21 | 17.32 | 12.89 | 10.46 | 5.33 | 4.36 | 0.49 | 0.97 |
| 2018 | 62.57 | 59.05 | 21.36 | 17.24 | 10.58 | 8.57 | 4.95 | 5.41 | 0.62 | 1.33 |
| 2019 | 64.39 | 60.52 | 20.67 | 14.98 | 9.99 | 7.84 | 4.47 | 4.34 | 0.28 | 0.69 |
| 2020 | 63.85 | 62.99 | 22.6 | 16.57 | 9.23 | 6.19 | 3.25 | 4.51 | 0.62 | 0.84 |
| 2021 | 65.48 | 63.39 | 19.15 | 15.7 | 9.43 | 6.41 | 4.16 | 4.39 | 0.37 | 1.27 |
| 2022 | 63.93 | 64.81 | 19.14 | 14.62 | 8.28 | 4.66 | 3.1 | 4.06 | 0.22 | 0.38 |

Figure S30. Prevalence of beta blocker, digitalis glycosides, calcium channel blocker, and antiarrhythmic drug therapy in the year following atrial fibrillation diagnosis, stratified by age, in patients with chronic obstructive pulmonary disease in UK primary care from 2010 to 2022

| **Beta blockers** | **Digitalis glycosides** |
| --- | --- |
| y-axis is on a different scale |  |
| **Calcium channel blockers** | **Antiarrhythmic drug therapies** |
|  |  |

Percent of patients prescribed

| Year | 50-59 years | 60-69 years | 70-79 years | 80-89 years | ≥ 90 years |
| --- | --- | --- | --- | --- | --- |
| 2010 | 40 | 45.77 | 38.59 | 27.52 | 25.22 |
| 2011 | 50 | 48.63 | 43.08 | 37.39 | 25.49 |
| 2012 | 45.95 | 56.51 | 49.44 | 42.52 | 33.7 |
| 2013 | 57.5 | 60.05 | 52.83 | 49.9 | 39.04 |
| 2014 | 68.52 | 63.33 | 55.52 | 51.14 | 43.62 |
| 2015 | 62.75 | 64.9 | 60.75 | 53.72 | 48.64 |
| 2016 | 76.92 | 69.44 | 61.07 | 56 | 50 |
| 2017 | 66.67 | 65.74 | 62.58 | 56.34 | 47.08 |
| 2018 | 62.12 | 68.57 | 63.26 | 57.01 | 48.69 |
| 2019 | 78.79 | 68.79 | 64.37 | 58.92 | 47.39 |
| 2020 | 81.48 | 72.7 | 67.53 | 59.88 | 38.6 |
| 2021 | 74.14 | 73.7 | 67.97 | 60.89 | 46.67 |
| 2022 | 81.67 | 73.95 | 66.77 | 61.02 | 50.2 |

Percent of patients precribed

| Year | 50-59 years | 60-69 years | 70-79 years | 80-89 years | ≥ 90 years |
| --- | --- | --- | --- | --- | --- |
| 2010 | 35.56 | 24.78 | 27.05 | 20.45 | 13.04 |
| 2011 | 23.81 | 23.63 | 23.06 | 17.27 | 11.11 |
| 2012 | 18.92 | 21.35 | 20.1 | 15.84 | 9.78 |
| 2013 | 27.5 | 19.85 | 18.2 | 15.9 | 8.22 |
| 2014 | 12.96 | 17.62 | 18.29 | 11.47 | 6.38 |
| 2015 | 15.69 | 16.78 | 13.99 | 9.73 | 8.64 |
| 2016 | 6.15 | 12.68 | 13.71 | 10.43 | 4.44 |
| 2017 | 15 | 15.2 | 12.1 | 10.01 | 7.78 |
| 2018 | 21.21 | 12.24 | 10.73 | 7.37 | 4.49 |
| 2019 | 9.09 | 8.75 | 10.83 | 7.18 | 4.78 |
| 2020 | 9.26 | 6.76 | 8.79 | 6.89 | 4.65 |
| 2021 | 8.62 | 10.68 | 8.91 | 6.39 | 3.7 |
| 2022 | 3.33 | 7.2 | 7.03 | 5.92 | 2.39 |

Percent of patients prescribed

| Year | 50-59 years | 60-69 years | 70-79 years | 80-89 years | ≥ 90 years |
| --- | --- | --- | --- | --- | --- |
| 2010 | 33.33 | 41.11 | 40.13 | 38.66 | 39.13 |
| 2011 | 26.19 | 37.36 | 39.37 | 35.53 | 39.22 |
| 2012 | 8.11 | 33.07 | 33.33 | 34.27 | 33.7 |
| 2013 | 25 | 30.75 | 29.16 | 29.18 | 34.93 |
| 2014 | 22.22 | 29.52 | 28.51 | 27.89 | 25.53 |
| 2015 | 25.49 | 24.5 | 26.45 | 24.25 | 20.45 |
| 2016 | 23.08 | 20.37 | 21.83 | 21.39 | 24.6 |
| 2017 | 16.67 | 20.56 | 21.03 | 21.23 | 22.57 |
| 2018 | 16.67 | 21.94 | 20.28 | 17.37 | 15.36 |
| 2019 | 10.61 | 19.09 | 18.43 | 16.91 | 11.3 |
| 2020 | 16.67 | 20 | 20.44 | 18.66 | 13.95 |
| 2021 | 20.69 | 17.19 | 19.28 | 15.75 | 13.33 |
| 2022 | 21.67 | 19.11 | 17.01 | 15.24 | 15.14 |

Percent of patients prescribed

| Year | 50-59 years | 60-69 years | 70-79 years | 80-89 years | ≥ 90 years |
| --- | --- | --- | --- | --- | --- |
| 2010 | 15.56 | 14.29 | 11.54 | 6.29 | 5.22 |
| 2011 | 33.33 | 15.38 | 9.34 | 6.96 | 0.65 |
| 2012 | 13.51 | 13.28 | 8.1 | 4.23 | 2.17 |
| 2013 | 15 | 12.59 | 6.78 | 5.54 | 2.05 |
| 2014 | 18.52 | 11.67 | 7.8 | 4.55 | 3.19 |
| 2015 | 11.76 | 9.05 | 6.06 | 4.07 | 4.09 |
| 2016 | 7.69 | 9.77 | 7.8 | 3.57 | 2.42 |
| 2017 | 16.67 | 8.78 | 5.54 | 2.58 | 1.17 |
| 2018 | 18.18 | 7.38 | 6.17 | 3.2 | 2.62 |
| 2019 | 12.12 | 6.56 | 5.05 | 3.09 | 0.87 |
| 2020 | 18.52 | 7.84 | 4.11 | 2.4 | 0.47 |
| 2021 | 6.9 | 9.64 | 5.31 | 1.92 | 1.48 |
| 2022 | 13.33 | 6.7 | 4.47 | 1.94 | 0.8 |

Figure S31. Prevalence of beta blocker, digoxin, calcium channel blocker and antiarrhythmic drug therapy use, and ablation procedures for atrial fibrillation, in the year following atrial fibrillation diagnosis, stratified by chronic obstructive pulmonary disease (COPD) exacerbation in the prior year, in patients with COPD in UK primary care from 2010 to 2022

Percent of patients prescribed

| Year | Beta blockers no exacerbation | Beta blockers exacerbation | Digitalis glycosides no exacerbation | Digitalis glycosides exacerbation | Calcium channel blockers no exacerbation | Calcium channel blockers exacerbation | Antiarrhythmics no exacerbation | Antiarrhythmics exacerbation | Ablation no exacerbation | Ablation exacerbation |
| --- | --- | --- | --- | --- | --- | --- | --- | --- | --- | --- |
| 2010 | 39.29 | 30.5 | 33.11 | 46.14 | 21.62 | 25.64 | 10.04 | 9.5 | 0.97 | 0.5 |
| 2011 | 46.16 | 35.66 | 32.68 | 42.1 | 17.5 | 23.04 | 8.93 | 9.71 | 0.8 | 0.71 |
| 2012 | 49.88 | 43.59 | 27.6 | 39.08 | 15.07 | 20.87 | 6.26 | 7.98 | 0.63 | 1.05 |
| 2013 | 56.34 | 48.48 | 22.79 | 35.86 | 14.21 | 19.91 | 7.06 | 7.14 | 1.36 | 0.87 |
| 2014 | 57.62 | 51.95 | 22.94 | 32.48 | 11.67 | 17.38 | 7.38 | 6.85 | 0.63 | 0.87 |
| 2015 | 60.68 | 55.54 | 18.76 | 30.09 | 10.5 | 14.13 | 6.11 | 5.38 | 0.72 | 0.8 |
| 2016 | 59.96 | 59.99 | 16.8 | 25.69 | 9.96 | 12.79 | 5.6 | 6.62 | 0.9 | 0.68 |
| 2017 | 61.02 | 58.41 | 17.2 | 24.45 | 9.49 | 13.22 | 4.01 | 5.42 | 0.51 | 1 |
| 2018 | 62.14 | 59.07 | 15.35 | 22.39 | 7.1 | 11.59 | 5.22 | 5.2 | 0.91 | 1.13 |
| 2019 | 61.66 | 62.53 | 13.13 | 21.08 | 6.87 | 10.38 | 4.36 | 4.42 | 0.55 | 0.49 |
| 2020 | 64.4 | 62.33 | 16.29 | 21.88 | 6.03 | 8.88 | 3.62 | 4.33 | 0.6 | 0.88 |
| 2021 | 64.89 | 63.55 | 13.65 | 21.86 | 6.02 | 9.96 | 3.96 | 4.72 | 0.69 | 1.12 |
| 2022 | 65.72 | 62.92 | 14.48 | 18.95 | 4.44 | 8.25 | 3.64 | 3.68 | 0.4 | 0.2 |

Figure S32. Percent of patients prescribed long-acting muscarinic antagonists or long-acting beta agonists alone or in combination, in the 3 months prior to and the three months after atrial fibrillation diagnosis in patients with chronic obstructive pulmonary disease in UK primary care from 2010 to 2022

Percent prescribed

|  | Before AF | After AF |
| --- | --- | --- |
| LAMA | 40.1 | 38.4 |
| LABA | 4 | 3.3 |
| LABA + LAMA | 5.8 | 5.7 |
| LABA + ICS | 46.1 | 43.2 |
| LABA + LAMA + ICS | 6.6 | 7.4 |

**Abbreviations:** LAMA, long-acting muscarinic antagonists; LABA long-acting beta agonists, ICS, inhaled corticosteroid.
